# The Expression of a Novel Mitochondrially-Encoded Gene in Gonadic Precursors May Drive Paternal Inheritance of Mitochondria

**DOI:** 10.1371/journal.pone.0137468

**Published:** 2015-09-04

**Authors:** Liliana Milani, Fabrizio Ghiselli, Andrea Pecci, Maria Gabriella Maurizii, Marco Passamonti

**Affiliations:** Department of Biological, Geological and Environmental Sciences, University of Bologna, Bologna, Italy; RIKEN Advanced Science Institute, JAPAN

## Abstract

Mitochondria have an active role in germ line development, and their inheritance dynamics are relevant to this process. Recently, a novel protein (RPHM21) was shown to be encoded in sperm by the male-transmitted mtDNA of *Ruditapes philippinarum*, a species with Doubly Uniparental Inheritance (DUI) of mitochondria. *In silico* analyses suggested a viral origin of RPHM21, and we hypothesized that the endogenization of a viral element provided sperm mitochondria of *R*. *philippinarum* with the ability to invade male germ line, thus being transmitted to the progeny. In this work we investigated the dynamics of germ line development in relation to mitochondrial transcription and expression patterns using qPCR and specific antibodies targeting the germ line marker VASPH (*R*. *philippinarum* VASA homolog), and RPHM21. Based on the experimental results we conclude that both targets are localized in the primordial germ cells (PGCs) of males, but while VASPH is detected in all PGCs, RPHM21 appears to be expressed only in a subpopulation of them. Since it has been predicted that RPHM21 might have a role in cell proliferation and migration, we here suggest that PGCs expressing it might gain advantage over others and undertake spermatogenesis, accounting for RPHM21 presence in all spermatozoa. Understanding how foreign sequence endogenization and co-option can modify the biology of an organism is of particular importance to assess the impact of such events on evolution.

## Introduction

All sexually reproducing organisms arise from gametes, cells coming from meiosis and a differentiation process. The first representatives of this cell lineage to appear in the embryo are the primordial germ cells (PGCs), a generally small population of early segregating cells that are committed to germinal fate. Derivatives of PGCs in sexually mature organism differentiate into gametes, then gametes will fuse and set up a new individual in which PGCs are specified again.

Germ line establishment in the embryo reflects two classic and general ontogenetic models: epigenesis and preformation [1]. In the epigenesis specification mode, also called inductive or regulative mode, the PGCs arise later in embryogenesis from pluripotent stem cells induced to become the germ line by interactions with neighbouring cells. On the contrary, in the preformation mode, PGCs are specified by a specialized maternal cytoplasm, generally called “germ plasm”, that is asymmetrically partitioned during oogenesis and/or after fertilization. The components of the germ plasm are proteins, mRNAs, small RNAs, nuclear and mitochondrial ribosomes and mitochondria. The presence of these signals inside germ cells defines their fate. Germ plasm observed at the Transmission Electron Microscope (TEM) contains an electron-dense granulofibrillar material, called "nuage", that is often positioned near the nucleus and associated with mitochondria in a distinctive structure known as Balbiani body [2–4]. In male germinal cells, the nuage is represented by the chromatoid body, which is also typically associated with mitochondria [5,6]. In the Manila clam *Ruditapes philippinarum* (which is the object of this study), as in bivalve molluscs in general, the blastomere 4d is the precursor of the germ line, and, given the identification of specific markers in presumptive germ cells, it seems that preformation is widespread in this animal group [7–10].

### The *vasa* gene

The *vasa* gene, firstly characterized in *Drosophila* [11,12], codes for an ATP-dependent RNA helicase. The protein is a member of the DEAD (Asp-Glu-Ala-Asp) box protein family, that belongs to RNA helicase superfamily II, the largest family of RNA helicases [13]. The VASA protein functions in a broad range of molecular processes involving duplex RNA, such as promoting translation of important mRNAs involved in development (e.g. *nanos* and *gurken* in *Drosophila* [14,15]) and promoting germ line specification. The mechanism of action is still largely unknown, but there is evidence suggesting that VASA may operate mainly as chaperon that unwind local secondary structures to facilitate proper RNA folding and interactions with accessory proteins [16], as well as facilitating their translation [17]. During *Drosophila* oogenesis, VASA is localized to the posterior of the oocyte and it is part of the pole plasm together with the products of other genes ([18] and references therein); by gastrulation, it is detectable only in germ cells, and their strong cytoplasmic staining is maintained during embryogenesis in both male and females [19].

VASA is very conserved among metazoans [20] and many orthologues, which play a role in germ cell determination, have been identified at the protein level in several species (reviewed in [21]). The discovery of transcripts of a *Crassostrea gigas vasa* homolog (*Oyvlg*), led to the conclusion that VASA is present also in molluscs, and that it can be used as a germ line marker [7,8]. A VASA homolog was also documented in *Mytilus galloprovincialis*, at the transcript level [9], and in *R*. *philippinarum*, at the protein level [10]. Recently, in mouse, a VASA homolog has been localized not only in the cytoplasm, but also inside mitochondria [22].

### Mitochondria and germ plasm

As previously mentioned, the presence of mitochondria within the germ plasm is known since the first studies that used TEM to investigate the ultrastructure of this specialized cytoplasm. The presence of mitochondria was originally thought to be simply associated with ATP synthesis [23]. It was only later, with the direct evidence for the presence of 16S rRNA in the germ plasm of several species (*Drosophila* [24]; *Xenopus* [25]; *Hemicentrotus pulcherrimus* [26]), that the mitochondrial involvement in germ line development was unveiled. In particular, it was observed that mitochondrial ribosomes exit in the cytoplasm and take part in translation of proteins that are necessary for the determination of germ cells. It is important to underline that the contribution of mitochondria is not intended as standing-alone, but it is integrated with general pathways controlled by the nuclear genome. In fact, for example, in *Drosophila* the protein encoded by the nuclear gene *tudor* is necessary for the localization of the mitochondrial rRNA into the germ plasm [27]. Ultrastructural evidence for the emission of mitochondrial matrix and cristae in the cytoplasm was provided in the sea urchin *Anthocidaris crassispina* [28,29]. Mitochondrial contribution was observed also in mouse, where dense granules released by mitochondria was documented [4]. In *R*. *philippinarum*, putative mitochondrial ribosomes in germ plasm were observed [10], thus it is likely that these organelles play a role in germ line determination also in this species.

To further understand the functions of mitochondria, as well as their deep connection with germ line recurrent structures, it is fundamental to deal with their inheritance dynamics. The animal kingdom is united in this sense with a general rule: the strictly maternal inheritance (SMI) of mitochondria. While nuclear genome follows a biparental inheritance, the mitochondrial genome is transmitted typically only through the female lineage and, even though exceptions have been recorded in some organisms, they are considered rare and accidental events [30]. If SMI is the almost unique rule in the animal kingdom, the mechanisms by which it is achieved are very diverse, suggesting that SMI has evolved multiple times [31,32]. The only known exception is represented by several bivalve species. In these molluscs, the mitochondrial inheritance is doubly uniparental: maternal mitochondria (and their genome, named F-type mtDNA) are transmitted through females, while paternal mitochondria (with a different genome, named M-type mtDNA) are transmitted through males. The zygote receives both maternal (through the egg) and paternal (through the spermatozoon) mitochondria, but it will transmit only one type of mitochondrial genome to the next generation, depending on its sex. The rule of uniparentality is not violated (each sex transmits its own mtDNA), thus the system was named doubly uniparental inheritance (DUI) [33–36].

The molecular mechanism through which DUI takes place is still largely unknown, but a few models have been proposed (see [37] for a review); in particular it has been suggested that nuclear genes are involved in DUI and interact with the product of mitochondrial genes in determining the sex and mitochondrial inheritance pattern [38,39]. Interestingly, in the Manila clam *R*. *philippinarum* a novel open reading frame has been found in the Unassigned Region 21 (UR21) of the M-type mtDNA (*orf21* or *rphm21*, from *R*. *ph* *ilippinarum* male-specific *orf* *21*), that is not present in the F-mtDNA. This open reading frame is transcribed [40] and translated [41]. *In silico* analyses [41,42] are consistent with a viral origin of the *rphm21* gene, and this would be in agreement with the hypothesis that sperm mitochondria of DUI organisms gained the ability to escape degradation and invade the germ line in males, a process that can be reminiscent of a viral infection. Nonetheless, in-depth analyses are still necessary to unveil some aspects of DUI, such as the possible M-type mtDNA involvement in its own maintenance in male gonad during development, and maybe in sex differentiation. In other words, the focus should be on the comprehension of M-type mitochondria invasiveness features in male germ line.

The understanding of how new biological resources can be acquired naturally by foreign sequence endogenization and co-optation are of particular importance. Indeed, these kind of studies can be useful in synthetic biology, that aims to develop new functions/possibilities by exploiting existing molecules or molecular machineries. These studies are actually implementing the synthesis and modification of bacteriophages for generating virus-based tools to treat infectious diseases [43]. For example, the clustered regularly interspaced short palindromic repeats (CRISPR)-associated system (Cas), the bacterial immune mechanism that confers resistance against foreign nucleic acids, was used to insert DNA sequences into genomes to repair mutational diseases [44]. In this concern, it has to be mentioned that *rphm21* upstream region presents similarities with Cas sequences [41]. Viruses, or parts of them, are frequently integrated into the host genome, and viral sequence endogenization can be a rapid source of variability, as in the case of transposable elements [45]. These sequences are usually remnants of ancient virus infections [46], and since all species show these “fingerprints”, their study in different organisms, both model and non-model, are necessary to obtain an exhaustive scenario, allowing to move sounder hypotheses on how these insertion events can shape evolution.

In this paper, we exploited the DUI species *R*. *philippinarum* to investigate the dynamics of germ line development in relation to mitochondrial transcription and expression patterns, focusing on a male-inherited novel mitochondrial gene of putative viral origin (*rphm21*). The experimental design followed two steps: first we identified by Real-Time qPCR the developmental stage in which the first germ cell proliferation event occurs in juveniles, and compared the transcriptional dynamics of F-type and M-type mitochondrial targets with that of gametogenic adults; then we used specific antibodies to localize the *R*. *philippinarum* VASA homolog (VASPH) and RPHM21 in juvenile and adult specimens.dBased on the obtained results we conclude that male germ cells express both the proteins, from their first appearance in juveniles to their proliferation during gametogenesis of fully grown adults, but while VASPH was detected in all visible germ cells, RPHM21 appeared to be expressed only in a subpopulation of them. Since RPHM21 was previously detected in all spermatozoa (see [41]), we propose that the germ cells expressing it might gain advantage over others and preferentially undertake spermatogenesis.

## Material and Methods

The bivalve reproductive cycle involves a period of gametogenesis, a spawning season, and a resting phase during which gonads are absent; the start and duration of reproductive stages mostly depend on water temperature, but also on food availability, photoperiod and salinity [47]. In Italy, *R*. *philippinarum* undergoes sexual quiescence from October to the end of January, so gonadal development starts in February and reaches its maximum in May-June. The spawning season starts in May and ends in late September, when gonads are reabsorbed and sexual rest begins [48,49]. All the specimens used in this study were sampled in 2013 and 2014 in Goro (Centro Ricerche Molluschi, CRiM; Ferrara, Italy). We define “juveniles” the individuals approaching their first reproductive season (maximum shell length 1–25 mm), and “adults” larger individuals sampled in a more advanced gametogenic stage.

Given the absence of macroscopic morphological sexual dimorphism, sex in bivalves can only be assessed by checking gametes using light microscope examination of animal sections, as long as some traces of the reproductive tissue are present. *R*. *philippinarum* is a gonochoric species, its gonads are located inside the connective tissue, at the base of the foot, associated with the digestive tube and, at sexual maturity, they occupy a large part of the animal body appearing as a light uniform fluid tissue. The structural unit of the gonad is the acinus, a sort of sack delimited by undifferentiated germ cells originating gametes that, at maturity, fill the center of the acinus, the lumen (see Fig 2 in [39]).

Before sample processing, we sexed the adult specimens with an optical microscope, then a body portion (containing a vast majority of gametogenic cells, although with traces of intestine and connective tissue that are anatomically impossible to discard) was excised and processed for subsequent analyses.

Juvenile specimens were measured and subdivided in size classes. Because of small dimensions, the developing gonadal tissue could not be excised, so the whole body was processed. For the same reason juveniles could not be sexed by visual inspection of gonads, therefore sex was indirectly attributed by the presence/absence of M-type mtDNA transcripts (*cytb_M* and *rphm21*).

### Real-Time qPCR

A total of 57 juveniles and 32 gametogenic adults were analyzed by Real-Time qPCR. Five target genes were amplified: 18S ribosomal RNA (*18S*), *vasph*, cytochrome b M-type (*cytb_M*), *rphm21*, and cytochrome b F-type (*cytb_F*); each sample was run in duplicate, so a total of 890 samples were analyzed (plus negative controls and standard serial dilutions). After dissection, whole bodies of juveniles were directly processed for RNA extraction using TRIzol (Life Tecnologies; standard protocol), and quantified using NanoDrop ND-1000. Reverse transcription to cDNA was performed using the High Capacity cDNA Reverse Transcription Kit (Invitrogen). Total RNA was diluted and the reaction was performed with 1.5 ng in 20 μL, on a 2720 termocycler (Applied Biosystems). After quantification with Nanodrop, cDNA was stored at-20°C. A StepOnePlus Real-Time qPCR System (Applied Biosystems) was used to quantify the targeted transcripts using SYBR green chemistry (for protocol details see [50]). Specific primers were designed with Primer3 ([51]; S1 Table).

The *vasph* gene was chosen as a proxy of gonadic cell proliferation activity, while mitochondrial transcription was assessed through 3 targets: *i)* the F-type *cytb* gene (*cytb_F*), present in both males and females; *ii)* the M-type *cytb* gene (*cytb_M*), present in males; and *iii)* the *rphm21* gene, present in males as well. *cytb* was chosen because it is the main subunit of the respiratory chain Complex III, the only one in this complex being encoded by the mitochondrial genome: this makes it a suitable first approximation for the whole mitochondrial transcriptional activity. Moreover, the same gene was widely used in the literature for similar purposes, also in studies on DUI animals (see [50] and references therein). The activity of the lineage-specific novel mitochondrial gene *rphm21* is of central interest for this work, so it was included among the qPCR targets. The *18S* gene was chosen as internal control for normalization (see [50] and references therein), but in juveniles we found out that it was not uniformly transcribed across all the samples (see Results), so it could not be used as reference gene in a relative quantification: for this reason, a standard curve method (“absolute quantification”) was carried out. The amplicons of each target gene were obtained by PCR, loaded on an agarose gel, excised, purified with the Wizard SV Gel and PCR Clean Up System (Promega), quantified and diluted to a stock concentration of 10 ng/μl. For each target, the stock solution was used to prepare a 10-fold serial dilution (from 10^1^ to 10^6^ copies) that was run in triplicates to generate the standard curve. The quantification was obtained using the equations reported in [52].

In the 32 gametogenic adults, the transcription level of *18S* resulted to be uniform, thus it was used as reference gene, and the ΔΔCq relative quantification method [53] was applied.

A cluster analysis (S1 Fig) was performed using agglomerative hierarchical clustering procedure (Wardelative quantification method [53] was applied.e plots were produced with the ggtern R package (http://www.ggtern.com/home). In the ternary barycentric coordinate system the position of a point is specified as the center of mass (barycenter) of masses placed at the vertices of an equilateral triangle. Thus, in a ternary plot the proportions of the three represented variables sum to a constant. The advantage of ternary plots is that three variables can be plotted in a two-dimensional graph. Each point in these plots represents a different composition of the three variables, with the maximum proportion (100%) of each variable in each corner of the triangle, and the minimum proportion (0%) at the opposite line. Statistical analyses and plots were obtained using R v3.1.1.

### Immunological analyses

#### Antibody production

An anti-RPHM21 antibody previously produced and tested [41] was used to detect the male-specific mitochondrial protein RPHM21. To visualize VASPH protein, we utilized specific antisera produced in rabbit (Davids Biotechnologie). These antibodies were generated against two peptides (19 amino acids each) synthesized from the predicted amino acid sequence at the C-terminus of the protein (HDSDSGMAKALVKILTQAS and KFGGKDIRKGMPKTRDEGE, acronyms HDS and KFG, respectively). The peptides were chosen among those with a good score for epitope prediction (algorithm by Davids Biotechnologie). Other factors that we took into consideration for the choice were: 1) peptide position in the VASPH 3D structure: VASPH models were predicted with I-Tasser (http://zhanglab.ccmb.med.umich.edu/I-TASSER/; [54]), and then the 3D structures were visualized in Chimera 1.8.1 [55]; the more external and easily reachable targets were choosen; 2) amino acid sequence that mostly differentiates VASPH from PL10 (the most closely related DEAD-box RNA helicase), to avoid cross-reactions. To do that, we identified PL10 homolog in the *R*. *philippinarum* transcriptome [56]. PL10 gene sequence was translated and aligned using Mega 5 [57] with the previously characterized VASPH sequence [10]. The obtained antibodies were tested for immunoreactivity by ELISA with the immunogen peptides and were later purified by affinity chromatography (Davids Biotechnologie).

#### Antibody specificity: western blot

Adult clams used for western blot were collected in June, when *R*. *philippinarum* is in an advanced gametogenic stage. Male and female gonads were freshly dissected and homogenized (using an Ultra Turrax T25 Janke & Kunkel IKA-labortechnik) in a buffer containing 10 mM Tris-HCl, pH 7.5, 1 mM ethylene glycol- bis(2-aminoethyl ether)-N,N,N’,N’-tetraacetic acid (EGTA), 0.1% Sodium Dodecyl Sulfate (SDS). One protease inhibitor cocktail tablet (Complete Mini, Roche) and 1 mM PMSF were added to 5 mL of the homogenization buffer before the use to limit degradation. Then samples were centrifuged at 10,000 rpm for 10 min at 4°C. The supernatant was stored at-80°C. Proteins of gonadic extracts were quantified with Lowry method and then separated via 8.5% Sodium Dodecyl Sulphate—PolyAcrylamide Gel Electrophoresis (SDS-PAGE) [58]. Gonadic extracts were mixed with Laemmli Sample Buffer (LSB) 2X and the mixture was then boiled 5 min at 95°C. Several amounts of protein (15 μg, 20 μg, and 30 in an advanced gametogenic stage. Male and female gonads were freshly dissected and homogenized (using an Ultra Turrax T25 Janke & Kunkel IKA-labortechnik) in a buffer containing 10 mM Tris-HCl, pH 7.5, 1 mM ethylene glycol- bis(2-aminod processed for staining with Coomassie Brilliant Blue. For immunoblotting, proteins were electrically transferred onto nitrocellulose membranes (Amersham Hybond Blotting Membranes, Buckinghamshire, UK). To prevent non-specific protein binding, aspecific sites were blocked with 5% dried skimmed milk (Bio-Rad Laboratories, Hercules, CA, USA), 3% Bovine Serum Albumin (BSA), in Tris-Buffered Saline (TBS) with 0.1% Tween-20 (Sigma) (TBS-Tw), 1 h 30 min at room temperature (RT), and subsequently washed for 30 min with TBS-Tw at RT. Then we proceeded with the incubation of membranes with polyclonal primary antisera against VASPH (anti-HDS and anti-KFG). The antisera, produced in rabbit and based on the sequence of the *vasph* gene [10], were diluted 1:8,000 for anti-HDS and 1:30,000 for anti-KFG with TBS-Tw and incubated over night at 4°C, then for 1 h 30 min at RT. After rinsing for 30 min with TBS-Tw, we processed the membranes through incubation with goat anti-rabbit secondary antibody conjugated with horseradish peroxidase (Santa Cruz Biotechnology Inc., Santa Cruz, CA, USA) at the dilution of 1:5,000 for 1 h at RT. The membranes were washed for 30 min, then they were detected using ECL Western Blotting Detection Reagents (Roche) and exposed to Hyperfilm ECL (GE Healthcare). Photographic plates were then digitalized by scanning.

Considering the result of a previous western blot analysis with an anti-VASA-chicken homolog [10], the expected molecular weight for *R*. *philippinarum* VASA homolog (VASPH) was 65 kDa.

Controls were performed using the synthetic peptides used for the primary antibody production that were added to the antibody solution at a 10-fold concentration before the incubation in order to chelate by competition every antigenic site of the primary antibody. In this way it is kept from binding its target and the bands of interest are strongly attenuated.

#### Immunohistochemistry

We analyzed both gametogenic adults (2 females and 4 males) and juveniles (19 individuals). In adult specimens, portions of tissues were collected in the area of the digestive tube, since gonads are strictly associated with this structure; for juvenile individuals the entire body was collected, due to their small dimensions. Out of the 19 juveniles, we chose 13 individual of 6–7 mm and 6 individuals of 9 mm (S1 Table), because, according to the qPCR results, this size range corresponds to individuals that are starting their first gametogenesis (biological class B1 and early B2, see S1 Fig). Samples were fixed in a solution containing 3.7% paraformaldehyde, 0.1% glutaraldehyde, 80 mM K-PIPES, 1 mM MgCl_2_, 5 mM EGTA and 0.2% Triton X-100, pH 7, for 3 h 30 min. Then tissue was rinsed in Phosphate Buffered Saline (PBS) (128 mM NaCl, 2 mM KCl, 8 mM Na_2_HPO_4_, 2 mM KH_2_PO_4_), pH 7.2, for 1 h with changes every 15 min. Afterwards samples were embedded in 7% agar. Sections of 100–150 μm thickness, obtained using a Lancer Vibratome Series 1000, were post-fixed with increasing concentrations of methanol (50 to 100%), rehydrated in Tris Buffered Saline (TBS; 10 mM Tris-HCl, 155 mM NaCl), pH7.4, and processed as free-floating sections. Unreacted aldehydes were reduced with 70 mM sodium borohydride in TBS (pH 7.4) for a 1 h 30 min at RT, followed by rinses for 1 h and 15 min in TBS. Antigenic sites were unmasked with 0.01% Pronase E (Merck Millipore) in PBS for 18 min at RT. Sections were rapidly washed with PBS in order to stop digestion, then samples were permeabilized adding TBS-1%Triton and left over night at 4°C. Non-specific protein-binding sites in both adults and juveniles sections were blocked with 1% Normal Goat Serum (NGS) and 1% BSA in TBS-0.1%Triton (TBS-0.1%T), pH 7.4, for 1 h 30 min. Then some sections were incubated with anti-HDS or anti-KFG, diluted 1:8,000 and 1:30,000 respectively; other sections were incubated with anti-RPHM21 polyclonal antibody (anti-SKE [41]) diluted 1:8,000. Primary antibodies were diluted with a solution of 3% BSA in TBS-0.1%T, pH 7.4. The incubation lasted 72 h at 4°C, followed by washes with TBS-0.1%T for 26 h. After this phase, sections were incubated in the dark with the secondary antibody [1:400 polyclonal goat anti-rabbit Alexa Fluor 488 (Life Technologies, Carlsbad, USA) in 1% NGS and 1% BSA in TBS-0.1%T, pH 7.4] for 32 h at 4°C. After washing 24 h with several changes in TBS-0.1%T pH 7.4, a nuclear counterstaining was performed with 1 μM TO-PRO-3 nuclear dye (Life Technologies, Carlsbad, USA) in PBS (pH 7.2) for about 10 min in the dark at RT, then the dye was washed in PBS and 30 min in TBS-0.1%T pH 7.4. All the immunostained sections were mounted in anti-fade medium [2.5% 1,4-diazabicyclo[2.2.2]octane (DABCO; Sigma), 50 mM Tris (pH 8) and 90% glycerol]. Slides were stored horizontally at 4°C in the dark. Imaging was recorded with a confocal laser scanning microscope (Leica confocal SP2 microscope), using Leica software. Controls were performed using samples in which the first antibody was replaced with 1% normal serum in TBS-1%T.

## Results

### Transcription level quantification identified three “biological classes” in juveniles and highlighted complex mitochondrial transcription patterns

In juveniles, the qPCR quantification cycle (Cq) of both the nuclear targets decreased exponentially from size-class S0 to S4 (from 1 to 15 mm, see S1 Fig and S3 Table) then reaching a plateau, meaning that the quantity of the nuclear targets increases with shell size, as expected. For this reason, the *18S* gene could not be used as reference gene to compare transcription levels across juvenile samples, and we adopted an absolute quantification method (see Material and Methods).

The cluster analysis (S2 Fig) performed on *vasph* Cq allowed the subdivision of juveniles in three groups, that we named s across juvenile samples, and we adopted an absolute quanoruvenile samples and the corresponding biological class is reported in S2 and S3 Tables.

In adults, the transcription level of *18S* was constant across all the analyzed samples, so it could be used as a reference gene, and a relative quantification method was applied (see Material and Methods).

**Fig 1:**
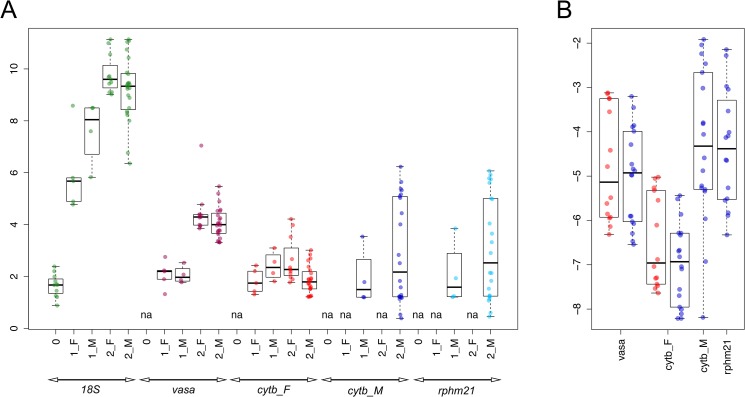
Transcription levels in juveniles (A) and adults (B). **(A)** 0 = class B0; 1 = class B1; 2 = class B2; F = Females; M = Males. y axis: Log_10_ copy number ("absolute quantification" with standard dilutions). **(B)** Red = Females; blue = Males. y axis: Log_10_ copy number relative to *18S* (relative quantification with *18S* as nuclear endogenous control).

In the biological class B0 (dimensional range 1–5 mm) the only detectable target was *18S*, whose transcription level grew progressively in classes B1 and B2 (Fig 1A). Also *vasph* showed an increased transcription from class B1 to class B2, with no difference between sexes. Between classes B1 and B2, *cytb_F* transcription seemed to increase in females and to decrease in males, even if the differences are not statistically significant. *cytb_M* showed a wide variance, especially in class B2, where its transcription level spans 6 orders of magnitude. Overall, there is no difference between transcription level in adult males and females, and the M-type mtDNA targets (*cytb_M* and *rphm21*) showed the highest variation (about 6 and 4 orders of magnitude, respectively) (Fig 1B).

**Fig 2:**
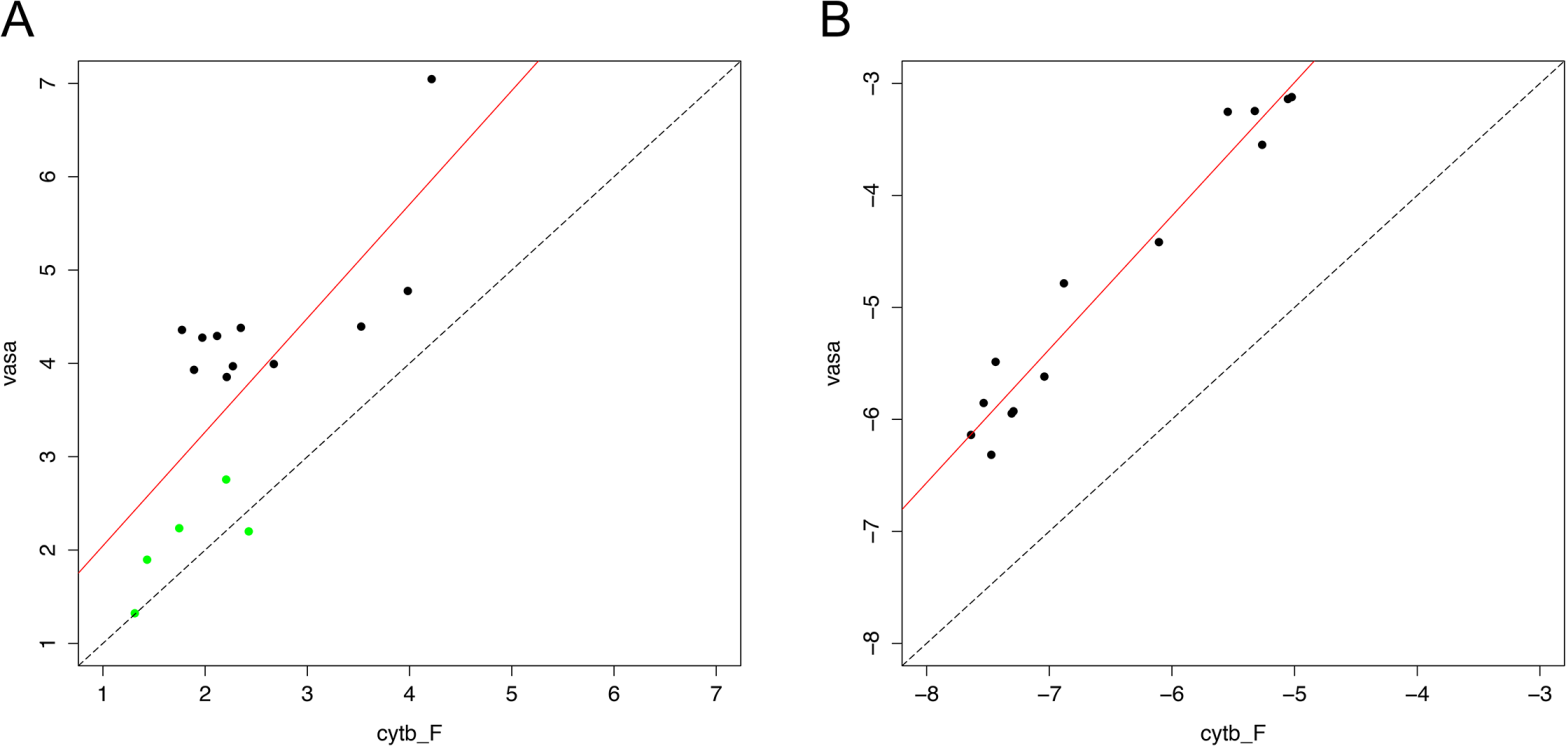
Transcription level correlations in juvenile (A) and adult (B) females. In females, *vasph* transcription resulted to be strongly correlated to mitochondrial transcription (statistics in S4 Table). Axes in A: Log_10_ copy number ("absolute quantification" with standard dilutions). Axes in B: Log_10_ copy number relative to *18S* (relative quantification with *18S* as nuclear endogenous control). In (A): green dots = B1 class; black dots = B2 class.

**Fig 3:**
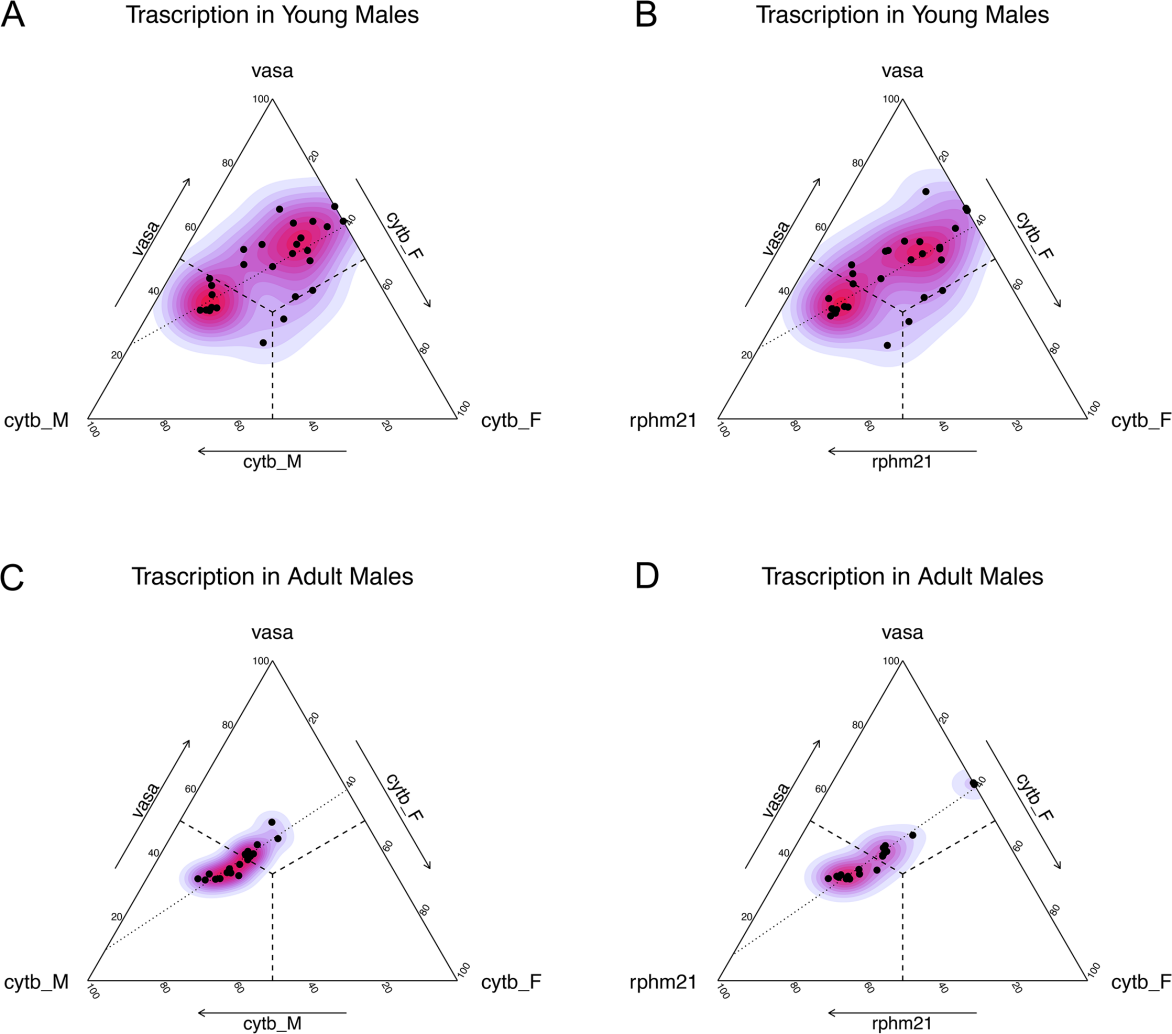
Transcription level in juvenile (A, B) and adult (C, D) males. **(A)** Transcription of *vasph*, *cytb_M* and *cytb_F* in young males. **(B)** Transcription of *vasph*, *rphm21* and *cytb_F* in young males. **(C)** Transcription of *vasph*, *cytb_M* and *cytb_F* in adult males. **(D)** Transcription of *vasph*, *rphm21* and *cytb_F* in adult males. Color gradient: kernel density estimation of the distribution. Dashed lines: middle segments representing equal transcription of the targets indicated at the vertexes of the orthogonal side. The three middle segments meet in the barycenter (i.e. the point of equal transcription of the three targets). Dotted lines: smoothed conditional mean of the distribution.

In females, *vasph* transcription resulted to be strongly correlated to mitochondrial transcription, both in juveniles and adults (Fig 2, S4 Table). In males, *vasph* and *cytb_F* transcription levels showed a strong correlation in adults but not in juveniles, while both the M-type mitochondrial targets did not show any correlation with *vasph* (S3 Fig, S4 Table). In male samples, where multiple mitochondrial targets were quantified, we also analyzed the relationships among *cytb_M*, *rphm21* and *cytb_F* (Fig 3, S4 Fig, S4 Table). The two M-type targets showed strong correlation both in juveniles and in adults (S4 Fig, S4 Table); *cytb_F* in juvenile males resulted to be negatively correlated with both the M-type targets, but such relationship did not hold in adults (S4 Fig, S4 Table).

### Production of specific anti-VASPH antibodies

**Fig 4:**
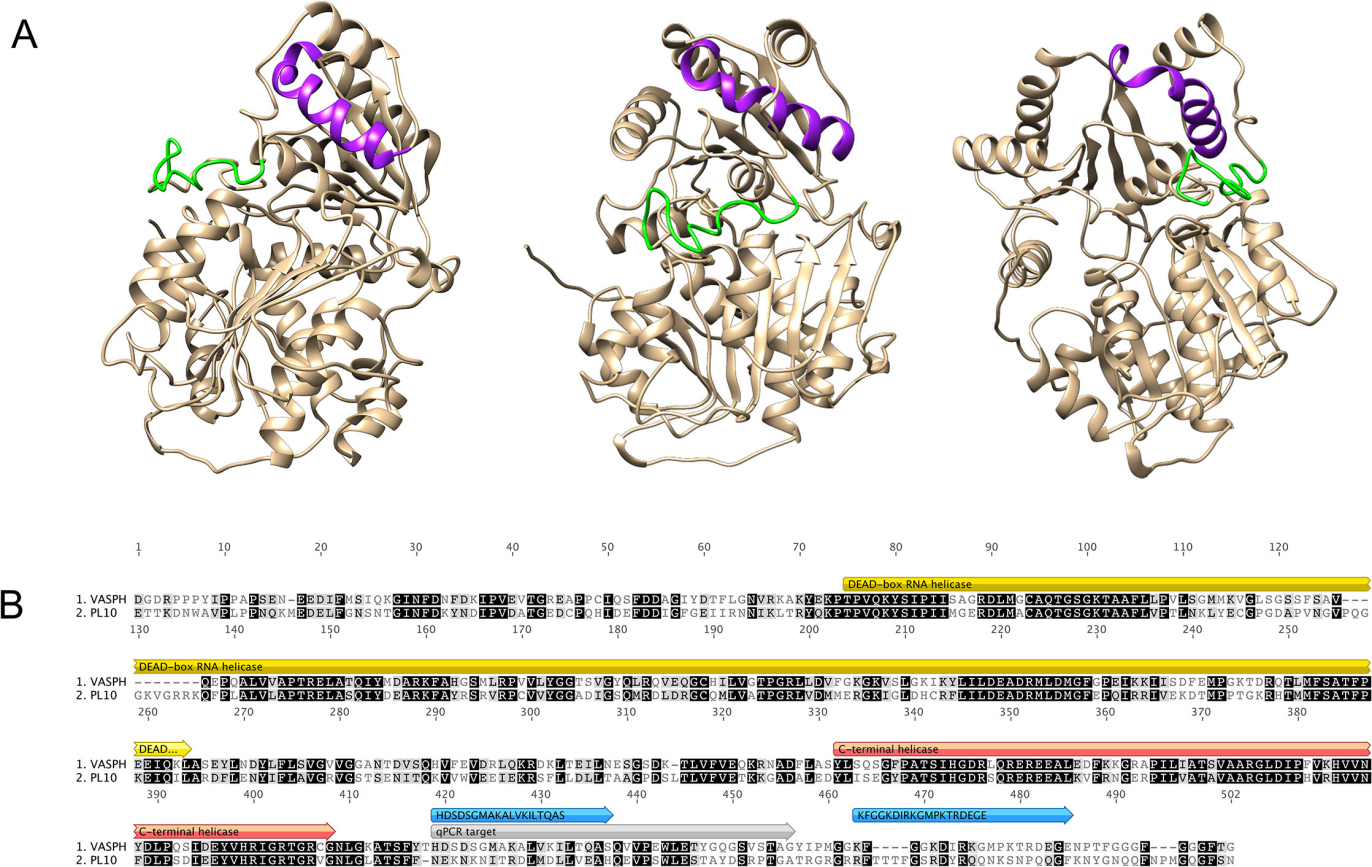
VASA homolog of *Ruditapes philippinarum* (VASPH). **(A)** VASPH structure model. Chimera 1.8.1 was used to model the protein structure. Peptides used for antibody production are highlighted: HDS in purple; KFG in green. **(B)** Alignment of VASPH and PL10 of *R*. *philippinarum*. Peptide location (blue), qPCR target (grey) and protein main domains (yellow and red) are highlighted.

The best protein structure predicted with I-Tasser had a C-score of-0.23. C-score is a confidence score for estimating the quality of predicted models by I-TASSER. It is calculated based on the significance of threading template alignments and the convergence parameters of the structure assembly simulations. C-score is typically in the range of [–5,2], where a C-score of higher value signifies a model with a high confidence and vice-versa [59]. The predicted structure was visualized with Chimera (Fig 4A). The two peptides were chosen among those with a good score for epitope prediction (Davids Biotechnologie), localized more externally in the predicted protein structure, and whose amino acid sequence mostly differentiates VASPH from PL10 to avoid cross-reactions. The position of the peptides targeted by the antibodies is shown in Fig 4A (purple and green sections on the 3D structure predicted by I-Tasser) and Fig 4B (blue arrows over the amino acid sequence).

### Western blot using anti-VASPH antibodies detected two bands with different intensity in males and females

**Fig 5:**
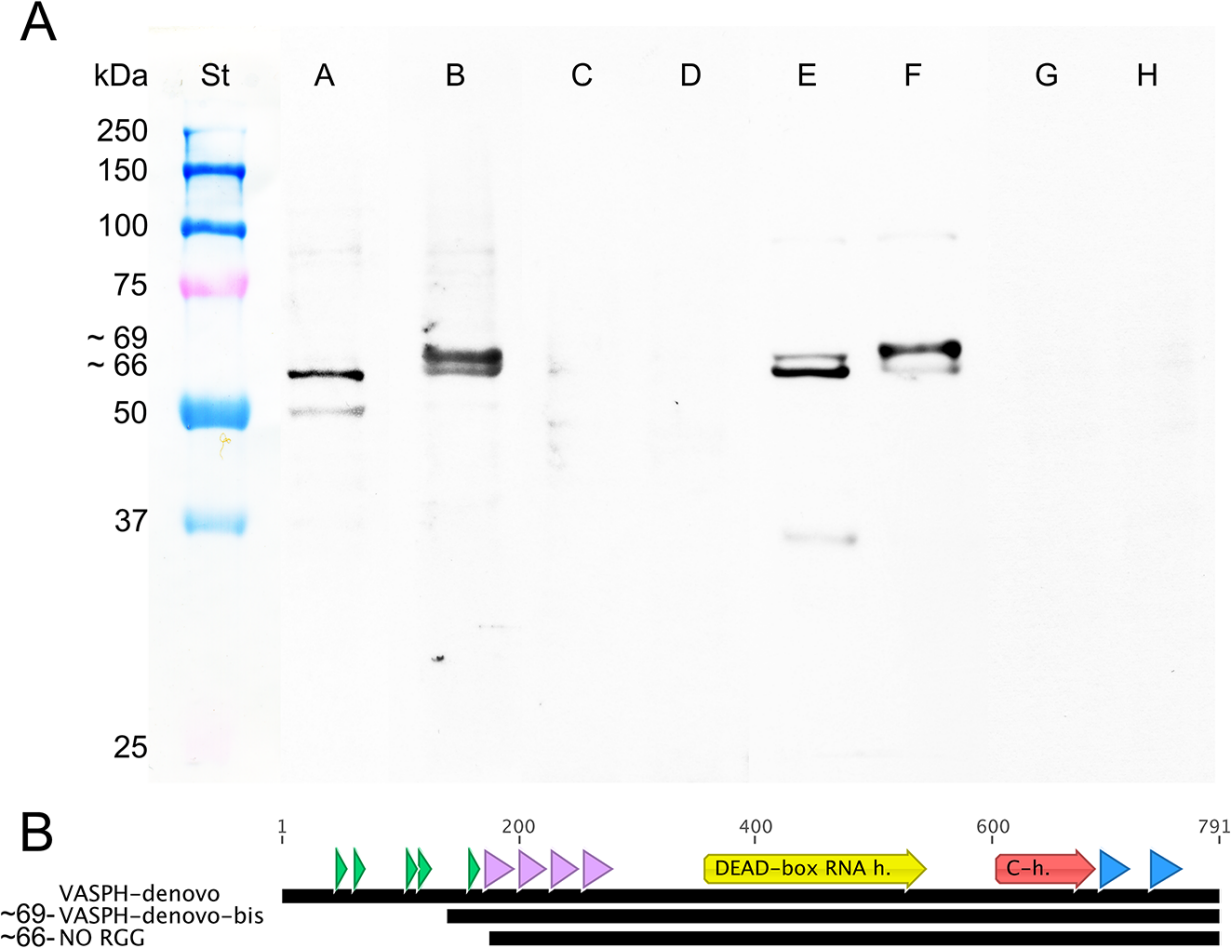
Detection of VASPH variants. **(A)** Anti-VASPH specificity: Western blots of ovary extracts (Oe) and testis extracts (Te) of adults. From left to right: St: protein standard. A: Oe/anti-VASPH-HDS. B: Te/anti-VASPH-HDS. C: Oe/anti-VASPH-HDS control. D: Te/anti-VASPH-HDS control. E: Oe/anti-VASPH-KFG. F: Te/anti-VASPH-KFG. G: Oe/anti-VASPH-KFG control. H: Te/anti-VASPH-KFG control. Western blots were obtained with the loading of 15 μg of homogenate per lane. **(B)** Different VASPH transcript assemblies highlighted different N-terminus of the protein. RGG motifs (green), zinc fingers (violet), protein main domains (yellow and red), and peptide location (blue) are highlighted.

Western blot was performed on gonad homogenates of gametogenic adults using the newly produced anti-VASPH (anti-HDS and anti-KFG antibodies) (Fig 5). According to the standards, in female homogenates, anti-HDS detected one band of approximately 66 kDa (lane A). This result is consistent with what obtained using an anti-VASA-chicken antibody [10]. In male homogenates, anti-HDS detected two close bands (lane B): the lower with the same weight of the band in the female sample, the other band with a slightly higher molecular weight (about 69 kDa). In both male and female lanes (lanes E and F, respectively), anti-KFG showed the same two bands detected with anti-HDS in the male sample. In the male lane, the highest band (69 kDa) was the most evident, conversely, in the female lane, the lowest band (66 kDa) was the strongest. The specificity of the antibodies was tested performing a western blot in which anti-HDS and anti-KFG antibodies were preincubated with a 10-fold molar excess (w/v) of the peptides against which they were produced. A significant reduction in the marking of the 66 and 69 kDa bands recognized by both the two antibodies was observed (lanes C, D and G, H in Fig 5). Fig 5B reports VASPH sequence obtained by different *de novo* assemblies [56]. The first assembly produced a longer sequence with 5 RGG motifs at the N-terminus [10]. The second assembly, performed to implement gene detection accuracy, produced a shorter sequence containing only one RGG in the same region. The rest of the protein was the same for both the assemblies.

### Protein immunolocalization in tissues showed VASPH and RPHM21 staining in primordial germ cells and their derivatives

All the analyzed specimens presented an epidermis surrounding a connective tissue with a lower cell density. The digestive tube, constituted by a single-cell layer wall and delimited by a basal lamina, is localized within the connective tissue. The digestive tube presents several branches that extend into the surrounding connective tissue. The flat, stretched nuclei of intestinal wall cells suggested a typical batiprismatic (columnar) intestinal epithelium. In gonads, germinal cells in different meiosis phases were distinguished by their size and nuclear chromatin morphology.

#### Germ cell marker: VASPH detection

**Fig 6:**
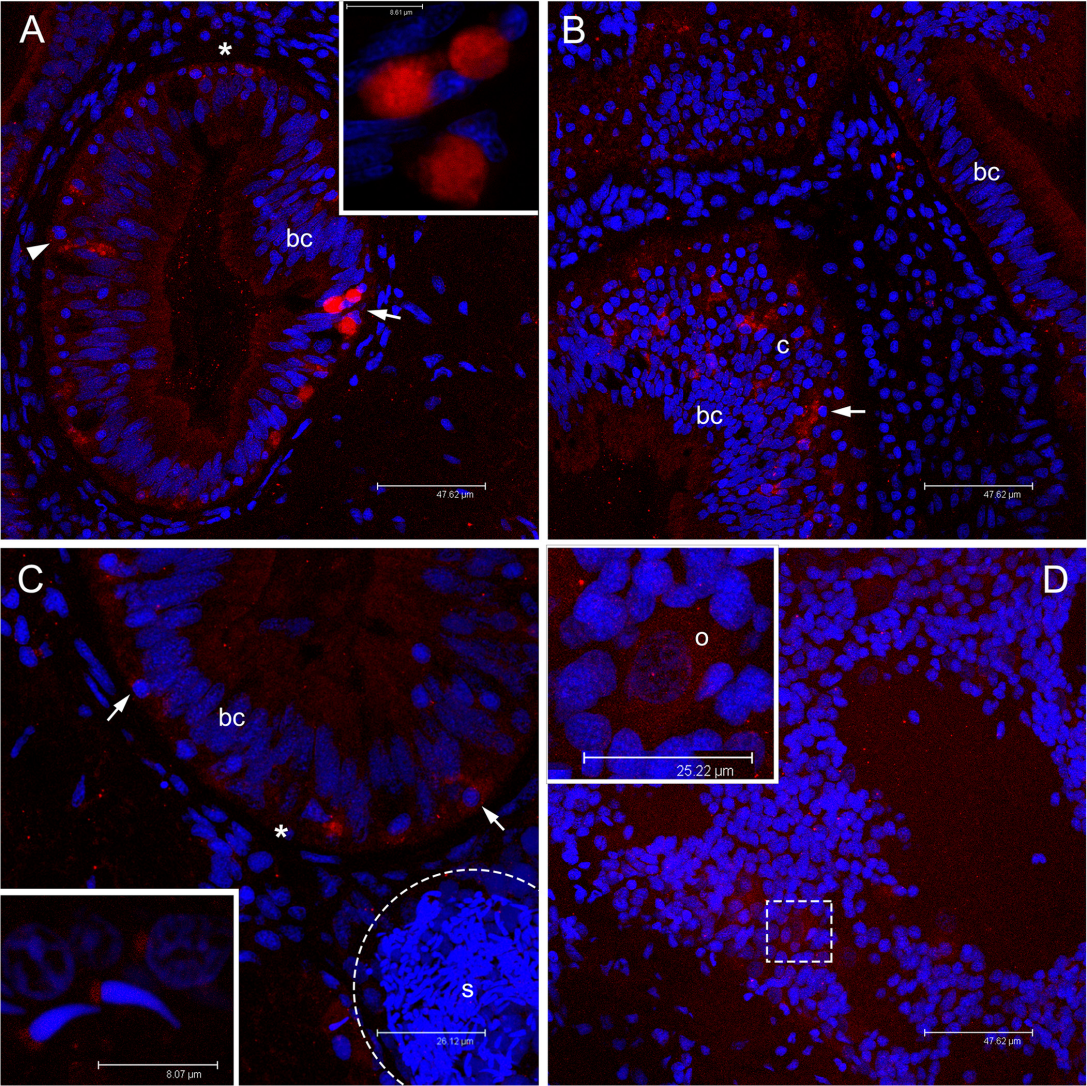
Immunolocalization of VASPH in germ cells of juveniles approaching their first reproductive season. **(A)** In juvenile clams, anti-VASPH highlighted many immunospots in germ cells with rounded nucleus (PGCs) localized in the thickness of intestinal epithelium, between unstained batiprismatic cells (bc) of the gut, and the basal lamina (asterisk). In some germ cells, the immunospots appear separated (arrowhead), while in other cells the spots aggregate at a side of the cell cytoplasm (arrow; magnification in the inset). Scale bar = 47.62 μm (inset scale bar = 8.61 μm). **(B)** Stained germ cells (arrow) are also visible in the connective tissue (c) between two intestinal loops. Scale bar = 47.62 μm. **(C)** Portion of young male section that shows an acinus (dashed circle) full of sperm (s) close to an intestinal loop in which some stained germ cells are present (arrows). The inset shows a magnification of two spermatozoa with a lightly stained mitochondrial midpiece and a spermatid (up right) with a big immunostained spot near the nucleus. Scale bar = 26.12 μm (inset scale bar = 8.07 μm). **(D)** Portion of young female section that shows early oocytes at different stage of development with a light VASPH staining in the cytoplasm. In the inset, a magnification of an early oocyte (o) showing few small granules. Scale bar = 47.62 μm (inset scale bar = 25.22 μm). Red: VASPH staining; blue: nuclear staining.

Juvenile clam sections treated with anti-HDS antibody or anti-KFG (VASPH detection) showed identical staining patterns in corresponding structures. Among cells that form the columnar epithelium, anti-VASPH binding revealed isolated stained cells (Fig 6A and 6C). These cells are significantly different from epithelial cells: they have a round nucleus and are often positioned at basal pole of the epithelium (Fig 6A and 6C). In some of these cells, the labelling appeared as big spots scattered in the cytoplasm, in others as a single cluster at a side of the cell cytoplasm (Fig 6A). Stained cells with the same nuclear morphology were also found interspersed in the connective tissue between intestinal loops (Fig 6B).

Compared to B1 juveniles (Fig 6C), B2 class showed a stronger anti-VASPH labelling (Fig 6A). Nonetheless, in both B1 and B2 specimens, groups of well differentiated spermatozoa, clearly recognizable from their nuclear morphology [60], were visible in isolated acini within the connective tissue near the gut (Fig 6C). Spermatozoa showed a clear labelling exactly in the posterior part of the spermhead (Fig 6C inset), where the mitochondrial midpiece, constituted by four-five mitochondria, is located [60]. In those acini, also some cells with big round nuclei with less condensed chromatin (likely round spermatids) showed a similarly localized labelling (Fig 6C inset, up right).

In sections of other specimens, instead of spermatozoa, faintly stained cells with a bigger cytoplasm mass than the surrounding connective cells were observed (Fig 6D). It is likely that they are female meiotic cells, considered to be part of simple-structured acini containing one or very few immature oocytes.

**Fig 7:**
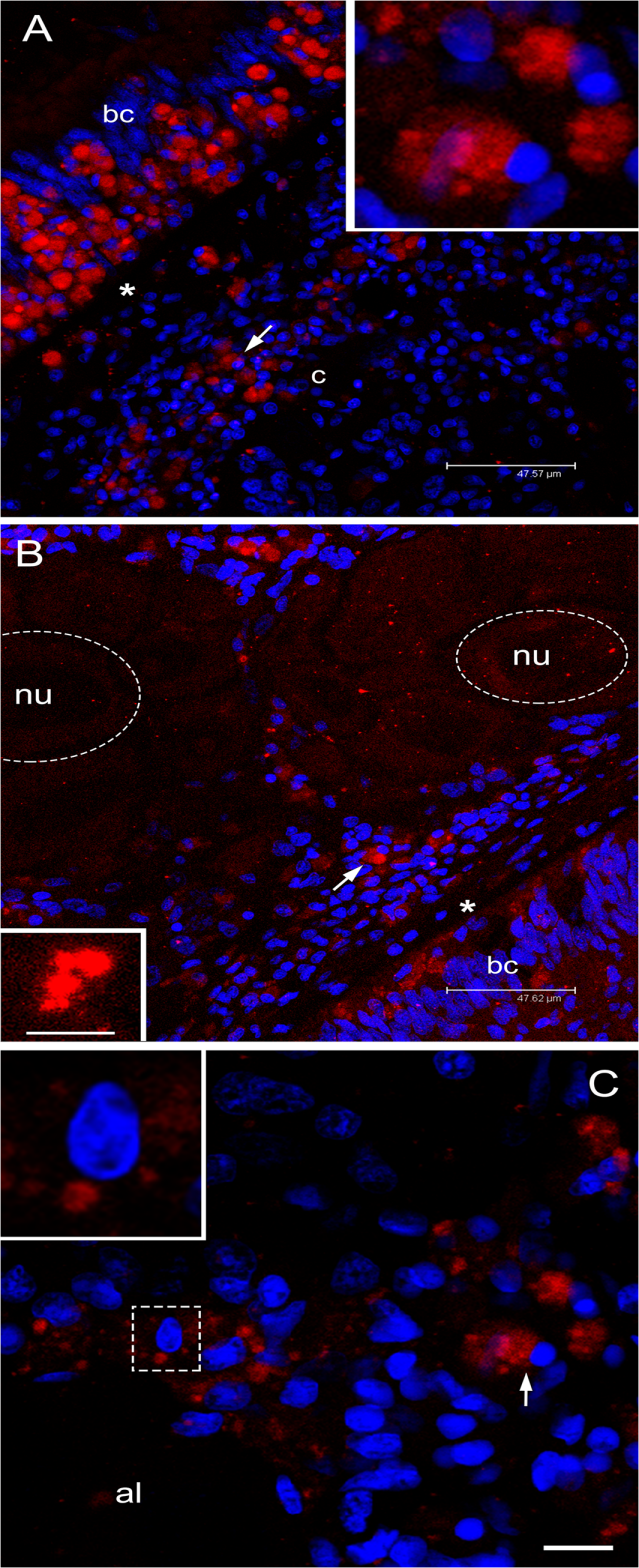
Immunolocalization of VASPH in germ cells of gametogenic adult females. **(A)** Section with a portion of intestine and connective tissue. Inside the intestinal epithelium, among batiprismatic cells (bc), a strong proliferation of VASPH stained germ cells (arrow) is observed. Many germ cells (arrow; magnified in the inset) have passed the basal lamina (asterisk) to the connective tissue (c). Scale bar = 47.57 μm. **(B)** In the connective tissue, in proximity of the intestine, germ cells (arrow) surround acini full of eggs (two eggs are highlighted with a dashed oval; nu: nucleus). In the egg, small stained granules are scattered in the cytoplasm (inset: granule magnification). Scale bar = 47.62 μm (inset scale bar = 4.87 μm). **(C)** At the periphery of an acinus lumen (al), very early oocytes of about 10 μm show big stained spots (one oocyte is magnified in the inset). Scale bar = 10.54 μm. Red: VASPH staining; blue: nuclear staining.

Sections of adult females treated with anti-HDS antibody or anti-KFG revealed an extensive proliferation of morphologically similar stained cells localized in the intestinal epithelium (Fig 7A), cells that in juveniles were more rare and often isolated (Fig 6). In all stained cells, the antibody labelling was present at a side of the cell cytoplasm (Fig 7A inset). Developed acini, containing mature eggs in the lumen and immature ones along the acinus wall, were detected close to the gut (Fig 7B). Some of these cells around the acini had the same strong labelling (Fig 7B). The staining was restricted to a smaller part of the cytoplasm in those we considered to be young oocytes attached to the acinus wall (Fig 7C inset), and even more reduced and scattered in more developed oocytes (Fig 7B inset).

**Fig 8:**
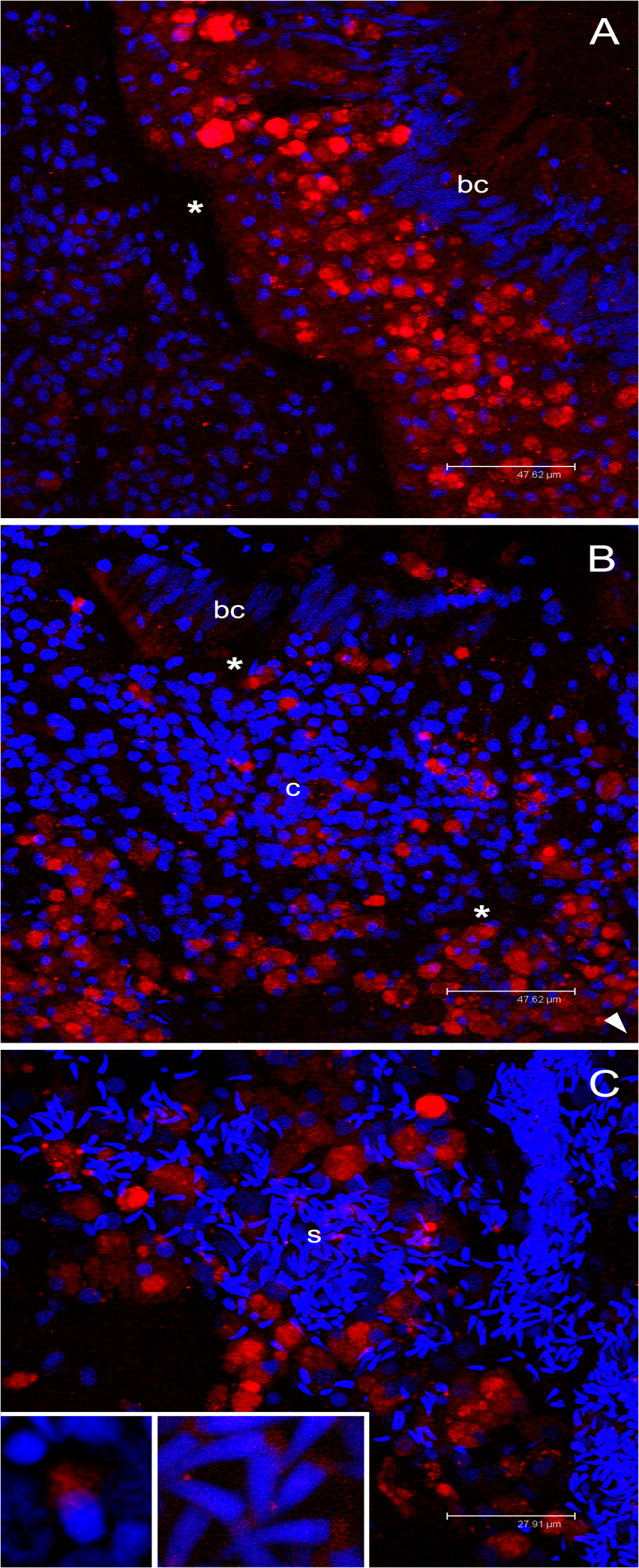
Immunolocalization of VASPH in germ cells of gametogenic adult males. **(A)** Strong proliferation of VASPH-stained germ cells in the intestinal epithelium at one side of the basal lamina (asterisk). Batiprismatic cells (bc) resulted VASPH-unstained. Scale bar = 47.62 μm. **(B)** Many stained germ cells in the connective tissue (c) between two intestinal loops (the arrowhead points to the position of other batiprismatic cells of the gut). Scale bar = 47.62 μm. **(C)** High magnification of a portion of male acinus showing many spermatozoa that fill the lumen. Inset on the left: spermatid with VASPH staining limited at the posterior part of the elongating nucleus. Inset on the right: several spermatozoa showing an even more reduced labelling in the mitochondrial midpiece. Scale bar = 27.91 μm. Red: VASPH staining; blue: nuclear staining.

As seen in females, also adult male sections treated with anti-VASPH showed an extensive replication activity of the same strongly stained and globose cells in gut (Fig 8A). Magnification of labelled cells revealed distinct spots in the cytoplasm, sometimes clustered in a bigger one (Fig 8). Near these sites of the intestine, developed acini were observed (Fig 8C). Strongly labelled cells, comparable to those localized in the gut wall, were detected in proximity of the acini (Fig 8C). Spermatozoa showed a faint staining at the level of the midpiece (Fig 8C inset), as seen in young males (Fig 6C inset).

#### Male-specific mitochondrial protein (RPHM21) detection

**Fig 9:**
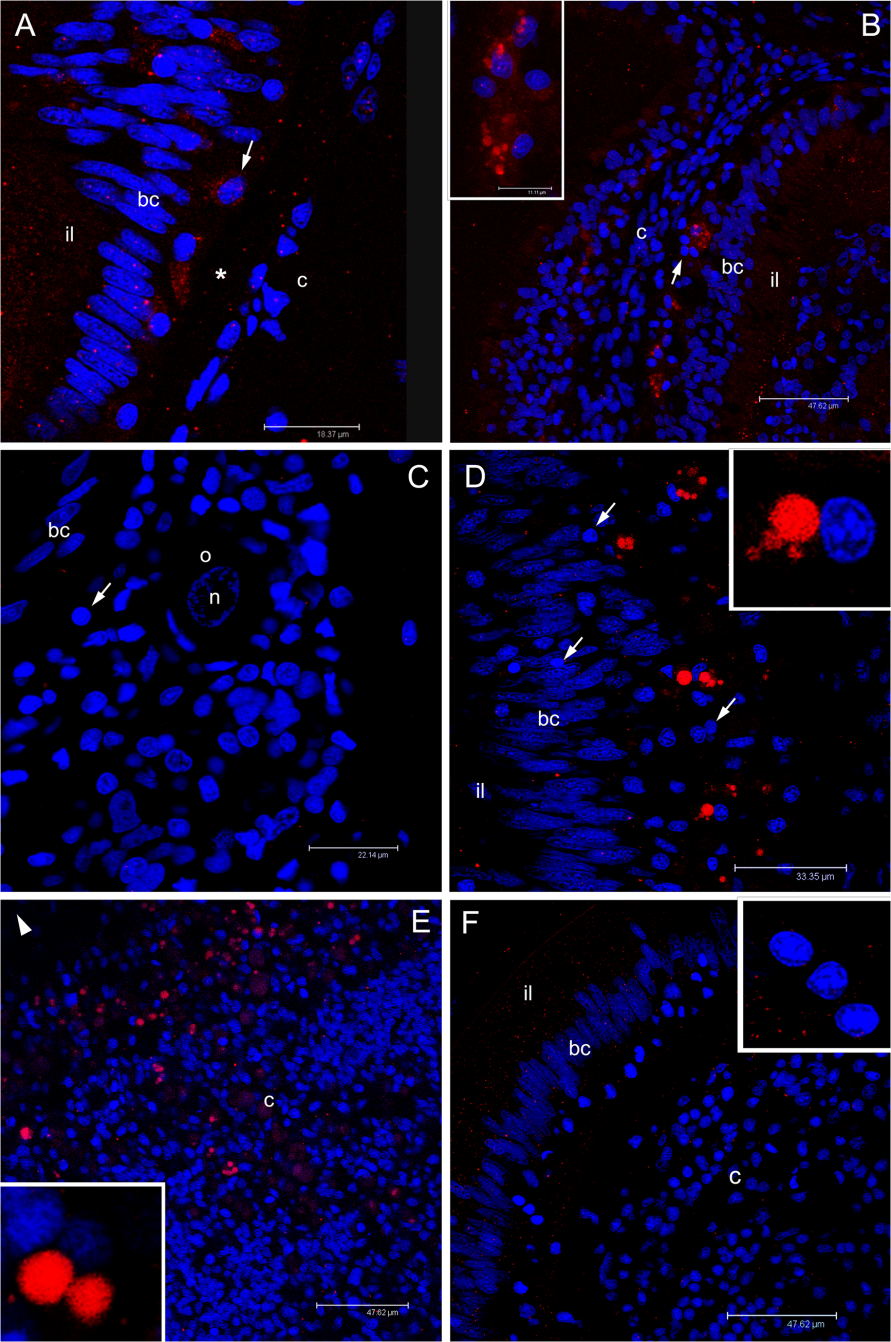
Immunolocalization of RPHM21 in germ cells. **(A)** In juveniles, the immunological reaction highlighted some rounded-nucleus cells (germ cells) with a diffused cytoplasmic RPHM21 staining (arrow) between unstained batiprismatic cells (bc) and the basal lamina (asterisk). Intestinal lumen = il; connective tissue = c. Scale bar = 18.37 μm. **(B)** In some sections of juvenile animals, germ cells are visible in the connective tissue (c) and show big stained spots (inset). Scale bar = 47.62 μm (inset scale bar = 11.11 μm). **(C)** In some female sections, simple acini, at the beginning of their organization, sometimes containing a single oocyte (o), were found. In these female sections, germ cells were visible (arrow) but no RPHM21 staining was present. n = oocyte nucleus. Scale bar = 22.14 μm. **(D)** Adult male section that shows RPHM21 stained germ cells close to batiprismatic cells (bc); some germ cells do not show any RPHM21 staining (arrow). Scale bar = 33.35 μm. **(E)** In male connective tissue, several RPHM21-stained germ cells are found (the arrowhead points to intestine position). The inset shows magnified RPHM21stained germ cells. Scale bar = 47.62 μm. **(F)** In adult female sections, no RPHM21-staining was detected in germ cells (magnified in the inset). Scale bar = 47.62 μm. Red: RPHM21 staining; blue: nuclear staining.

In some B1 and B2 sections, anti-SKE antibody (RPHM21 detection) showed a few labelled cells at the basal pole of the gut epithelium (Fig 9A). These cells were morphologically identical to the anti-VASPH stained cells (Fig 6). The same stained cells were also detected in the connective tissue (Fig 9B). Many sections showed single differentiating oocytes with no RPHM21 labelling (Fig 9C).

Also in adults, anti-RPHM21 immunoreaction generally followed the pattern of anti-VASPH antibodies: cells localized in the gut epithelium showed a strong labelling at one side of the cell cytoplasm, close to the nucleus (Fig 9D). Nonetheless, in males, while all these cells were anti-VASPH stained (Fig 8A), only a subpopulation of them appears to be anti-RPHM21 stained (Fig 9D). Stained cells were also detected in the connective tissue (Fig 9E) close to recognizable acini.

No RPHM21 labelling was detected in any of the round-nucleus cells in the gut wall of adult female sections (Fig 9F). Control on adult sections treated with secondary antibody (omitting the primary one) showed no labelling in the anatomical structures described above (S5 Fig).

## Discussion

### *vasph* transcription levels pinpoint the beginning of germ line proliferation

The first step of this work was to identify the beginning of germ line proliferation, that is the start of gonad formation. The sampled juveniles were approaching their first spawning season, and the observed boost in *vasph* transcription clearly indicates the beginning of the first germ cell proliferation. Each of the three biological classes, identified by the cluster analysis on qPCR data, includes individuals sharing a similar stage of gonad development (S1 and S2 Figs): *i)* B0 class showing no detectable *vasph* transcription, thus indicating a pre-proliferation stage; *ii)* B1 class showing increasing *vasph* transcription, thus indicating the beginning of gonad development; *iii)* B2 class showing an uniformly high level of *vasph* transcription, which indicates an established gametogenic phase. According to the present data, gametogenesis in *R*. *philippinarum* starts around 5 mm of shell length, and reaches its full capacity around 9 mm; we used this information to select samples for immunohistochemical analyses (see S3 Table).

### Transcriptional profiles in juveniles and gametogenic adults mirror early and late gamete differentiation stages

The second step of our experimental design was to characterize the mitochondrial transcriptional activity during germ line proliferation in juveniles, and in gametogenic adults. In females the transcription levels of *vasph* and *cytb_F* show a strong correlation both in juveniles (Fig 2A and S4 Table) and in adults (Fig 2B and S4 Table). This result is consistent with what has been observed in vertebrates (e.g.: human [61]; frog [62]; marsupial and monotremes [63]), where *vasa* is expressed from the multiplication (mitotic) stage of oogenesis (thus in oogonia) through the growth phase (thus in primary and secondary oocytes), and its activity is highly reduced in mature eggs. At the same time, mitochondria undergo an intense multiplication phase, being their biogenesis tightly linked to cell proliferation [64–66].

In males the situation is not as straightforward as in females, due to the presence of two mitochondrial lineages, with different activity, localization and biological role. As in oogenesis, during spermatogenesis *vasph* is active in the proliferative stages, that is from spermatogonia to spermatocytes II, and in spermatids, while its activity strongly decreases in mature spermatozoa. For what concerns mtDNA, in DUI animals, male gametes were shown to carry only M-type [52,67], so the F-type transcripts detected in male gonads are most likely produced by nurse and/or somatic cells. Actually, a specific localization of M-type transcriptional activity in spermatogenic cells and mature spermatozoa was also observed by *in situ* hybridization in *R*. *philippinarum* and *M*. *galloprovincialis* [41,68].

To better understand the complex relationship among the target genes in males, we visualized simultaneously the transcriptional activity of *vasph*, the F-type mtDNA target (*cytb_F*) and a M-type mtDNA target (either *cytb_M* and *rphm21*) (all visualized in Fig 3). Juveniles (Fig 3A and 3B) show a more variable pattern, most likely due to different stages of gonadal development. However, as highlighted by the kernel density estimation (color gradient), two main groups can be identified in males, representing individuals at early (upper right cluster) and more advanced (lower left) gametogenic stage. This subdivision is further supported by the observations in adults (Fig 3C and 3D), which show a more consistent transcriptional profile (i.e. with less variation) and a single cluster, in correspondence with the cluster of the more mature juveniles.

The patterns in males can be explained by the dynamics of gonad maturation. In early gametogenic juveniles (upper right cluster in Fig 3A and 3B) *vasph* and *cytb_F* are more transcribed than M-type mtDNA (*cytb_M* and *rphm21*): in this phase, the acini are forming or they have just begun the gametogenic phase, so the number of proliferating germ cells and mature spermatozoa is relatively small, explaining the low level of M-type transcripts. As the gametogenesis progresses (lower left cluster in Fig 3A and 3B), the M-type transcripts become more abundant, in respect to both *cytb_F* and *vasph*, accordingly with the increasing number of M-type-carrying cells. In adults the transcription of *cytb_F* and *vasph* is strongly correlated (Fig 3, S4 Table), while in juveniles the samples form two clusters reflecting the biological classes, but they do not show any correlation (S3 Fig, S4 Table). We think this could be due to the high transcriptional variance of both genes in juvenile samples. The two M-type mtDNA targets (*cytb_M* and *rphm21*) are strongly correlated in both juveniles (S4 Table) and adults (S4 Table), as can be seen in S4 Fig. This further supports the fact that mtDNA transcription levels are quite homogeneous, as a result of a polycistronic transcription, and that changes in expression are mostly due to post-transcriptional and post-translational regulatory activities (see a detailed discussion in [50]). In accordance with the dynamics of gonad development described above, the relationship between M-type and F-type transcripts in juvenile males is of negative correlation (see S4 Fig and S4 Table): in immature individuals and at the beginning of gonad development, the F-type is predominant, then the M-type takes over, as can be seen in adults (Fig 3, S4 Fig).

### Western blot and transcriptome analyses suggest the presence of VASPH protein variants

The western blots performed to test the specificity of the two produced anti-VASPH antibodies detected two close bands (around 66 and 69 kDa) (Fig 5A, lanes A,B,E and F) that could represent VASPH isoforms, a possibility proposed in literature for other VASA homologs [69–71]. Interestingly, the two identified bands showed an alternative expression pattern in males (Fig 5A lanes B,F) and females (Fig 5A, lanes A,E), consistent with the presence of a sex-specific splice variant: *vasa* isoforms originated via alternative splicing were found or inferred in several species (see for example [21]). Nevertheless, we did not find VASPH isoforms in our transcriptome data [56], therefore either we missed *vasa* splice variants, or the two different western blot bands are the result of post-transcriptional and/or post-translational modifications, a possibility reported for other species [21]. The different weight of the two bands can be due to the presence/absence of a RGG motifs. Protein isoforms lacking the N-terminal RGG/RG sequence exist, whose expression is restricted to a specific tissue (for example a testis specific expression, see [72]). We hypothesize a relationship between the N-terminus length and the presence of two VASPH bands, with the heavier isoform more expressed in the testis and the lighter isoform in the ovary. If we consider the protein without the N-terminal RGG motif the calculated protein weight (http://www.bioinformatics.org/sms/prot_mw.html) would be approximately 66 kDa (65.61 kDa). Considering only one RGG motif, as in VASPH from the new assembly (see Fig 5B and Results), the weight would be around 69 kDa (68.77 kDa). Although reports of variants lacking RGG/RG sequences are emerging, the regulation of RGG/RG sequences post-translationally modified by arginine methylation remains the most common mechanism of modulating the function of the RGG/RG motif [73].

### Germ line localization and developmental dynamics

We stained *R*. *philippinarum* VASA homolog (VASPH) to visually identify germ line and to assess whether or not VASPH is co-expressed with the male-specific mitochondrial protein RPHM21, a novel mitochondrial element of predicted viral origin supposed to be linked to male gonad development in the Manila clam [41]. We found that in the DUI clam *R*. *philippinarum* the expression of RPHM21 is male specific and that the expression of VASPH and RPHM21 occurs in the same cells, namely the PGCs.

Since appeared quite clear from transcription analysis that germ cell proliferation starts in the biological class B1, we performed immunohistological analysis on B1 and early B2 specimens, as well as on adults for comparison. PGCs appear to be very scarce in B1 juveniles and increase in number in B2 specimens (Fig 6A and 6C, respectively). In some cases, acini containing morphologically mature spermatozoa were found both in B1 and B2 individuals (Fig 6C). Although both males and females were reported to reach sexual maturity for the first time between 15 and 20 mm of size [74], some gametogenic clams between 5 and 10 mm in shell length were recorded [75]. Our data support the starting of proliferation of germinal cells around that dimensional range (specifically > 6 mm; S3 Table).

PGCs multiply considerably in gametogenic adults approaching the spawning season, and show a strong VASPH labelling (Figs 7 and 8). In females, as in males, VASPH-stained cells with round nucleus were also localized around the acinus wall (Figs 7 and 8); they probably represent gonia or cells at initial stage of gametogenesis. An attenuation of the labelling was observed in oocytes during their ongoing differentiation: in young oocytes of about 10 μm of diameter, we found a recognizable VASPH-stained spot (Fig 7C). Investigation by TEM showed that in early developmental stages of *R*. *philippinarum* gametes the nuage was visible in both the Balbiani body and the chromatoid body [10]. As reported above, VASA is a component of the nuage [76,77], thus the above-mentioned big VASPH-stained spots flanking the nucleus in young oocytes are most likely nuage material (Fig 7C). This material spreads and diffuses in the oocyte cytoplasm during its development, and VASPH distribution inside the oocytes suggests its presence in aggregates (Fig 7B, inset). In vitellogenic eggs the aggregates were generally no more recognizable (also with TEM, see [10]).

In male acini, the VASPH staining appeared to decrease during germ cell maturation showing a smaller labelled spot near the nucleus in spermatids (Fig 8C, inset on the left); in spermatozoa the VASPH labelling, extremely reduced, is observable in the few cytoplasm remained after spermiohistogenesis (Fig 8C, inset on the right). In this case, anti-VASPH staining could account for the presence of the protein in the very little cytoplasm present in the midpiece, where the chromatoid body is displaced in many animals [77], but also inside the mitochondria that constitute the midpiece, as recently reported in mouse [22].

### A proposed *rphm21* role in male germ cell establishment

As expected, anti-RPHM21 staining was localized in male germ cells (Fig 9A, 9B, 9D and 9E), while no signal was detected in female samples (Fig 9C and 9F). Being RPHM21 encoded by M-type mtDNA, its absence in females is expected and consistent with previous results obtained on mature gametes [41], where neither the transcript nor the protein were found in ovaries. That said, the presence of RPHM21 also in sperm nuclei might indicate the existence of a nuclear copy of the gene, and that the protein absence in female mature gonads might be the result of a tissue and/or stage specific expression. However, according to the results here reported, RPHM21 appears to be absent also from females at different gametogenic stages, and from both somatic and germ cells, thus strengthening the hypothesis of an exclusive mitochondrial origin of this protein, that would be both retained in the organelle and exported.

Anti-RPHM21 immunolabelled granules observed in the cytoplasm (Fig 9A and 9B) may represent aggregation of mitochondria in which the protein is concentrated. In adult males, the labelling in the PGCs is more compact and stronger, suggesting the exportation of the protein RPHM21 to the cytoplasm too (Fig 9D and 9E). The granules labelled with anti-RPHM21 as well as those labeled with anti-VASPH are localized at one side of the cell, suggesting that both proteins could be components of the germ plasm.

**Fig 10:**
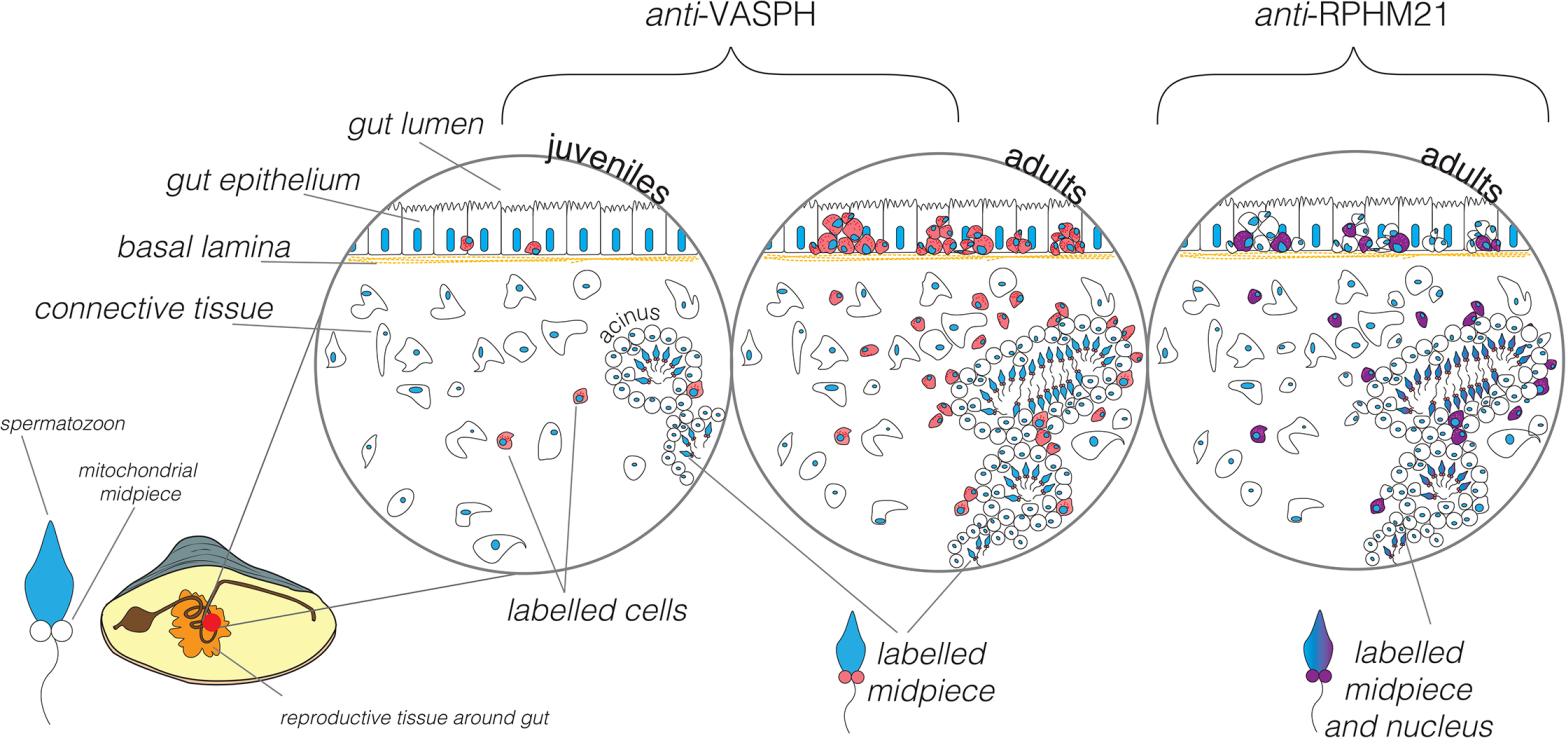
Scheme of RPHM21 and VASPH immunolocalization in germ cells during male gonad formation. VASPH expression (in red): in juvenile males (first circle on the left) few, stained PGCs are localized in the intestine among batiprismatic cells, and other stained germ cells are found in the connective tissue or around the few simple-structured acini localized in the connective tissue. In gametogenic males (circle in the middle) PGCs are massively proliferating among batiprismatic cells and are strongly immunostained. In mature male acini full of spermatozoa, a diffused VASPH-staining is present in the spermatogenic cells located near the acinus wall (see also [10]). Spermatozoon midpiece appears slightly stained. RPHM21 expression (in violet): only a subpopulation of PGCs located in the intestinal epithelium appears to express RPHM21, other PGCs, recognizable for their round nucleus, result completely negative to the RPHM21 staining. Some cells with a weak RPHM21 labelling (spermatogenic cells) are recognizable in the acinus wall [41]. RPHM21 is expressed in mature spermatozoa localized in the acinus lumen, both in mitochondria and the nucleus [41]. The staining of both factors (VASPH and RPHM21) is almost always condensed in a big cluster at one side of the cell cytoplasm.

Comparing the protein labelling pattern in adult males, VASPH appears to be present in all the recognizable PGCs, while RPHM21 seems to be localized only in a subpopulation of PGCs, since many germ cells do not show any RPHM21 staining (Fig 10). On the other hand, previous analyses performed on male mature gonads [41], showed that RPHM21 transcript and protein were present in all spermatozoa (unlabelled spermatozoa were never found).

This leads to a working hypothesis: what if germ cells carrying M-type mtDNA and expressing RPHM21 gain some sort of advantage during gametogenesis over the germ cells not expressing it? We are not able to confirm this scenario at the moment, but this advantage might consist of a faster or more efficient proliferation of the cells whose mitochondria express RPHM21.

If this hypothesis was proved to be true, some roles would be conceivable for RPHM21. For example RPHM21 might act in the process in which VASPH is an upstream regulator as germ line determinant, driving a spin-off pathway that leads to transmission of M-type mitochondria through male gametes. Alternatively, RPHM21 might be involved in a process of active elimination of germ cells not expressing it, something that would resemble a meiotic drive. The process might also be quantitative, namely the probability of a germ cell to reach the spermatozoon stage would depend on the amount of *rphm21* product expressed.

So far, the data in our possession would indicate a quite strict selection in favor of M-type-carrying gametes, but an extensive and deep sequencing of sperm mtDNA is needed to better clarify this point, which is fundamental for understanding how DUI works and specifically how spermatozoa transmit only M-type mtDNA. The here proposed mechanism could represent the Checkpoint #3 hypothesized in a previous work [52], in which the male germ line gets its homoplasmy, a fundamental feature of DUI systems: if sperm mitochondria contained also F-type mtDNA on a regular basis, DUI would collapse (see [37] for a detailed discussion).

## Supporting Information

S1 Fig Quantification cycles (Cq) of nuclear targets in juvenile size classes. (PDF)

S2 Fig Cluster analysis of juveniles based on *vasph* transcription level: definition of the biological classes (B0, B1, and B2). (PDF)

S3 Fig Relationships between the transcription level of *vasph* and mitochondrial targets. (PDF)

S4 Fig Relationships between the transcription level of mitochondrial targets. (PDF)

S5 Fig Immunohistochemical negative controls. (PDF)

S1 Table Primers used in Real-Time qPCR. (PDF)

S2 Table Juvenile sample for qPCR: subdivision in size and biological classes. (PDF)

S3 Table Summary of the juvenile samples used for the different analyses. (PDF)

S4 Table Transcription correlation statistics. (PDF)
