# Supplementary material for: The Expression of a Novel Mitochondrially-Encoded Gene in Gonadic Precursors May Drive Paternal Inheritance of Mitochondria
Source: PLoS One. 2015 Sep 4;10(9):e0137468. doi: 10.1371/journal.pone.0137468 (PMC4560408; doi:10.1371/journal.pone.0137468)

S1 Fig. Quantification cycles (Cq) of nuclear targets in juvenile size classes.

(A) Cq variation of *18S*; axes: Log10 copy number ("absolute quantification" with standard dilutions).

(B) Cq variation of *vasph* (*vasa* homolog of *Ruditapes philippinarum*); axes: Log10 copy number relative to *18S* (relative quantification with *18S* as nuclear endogenous control).

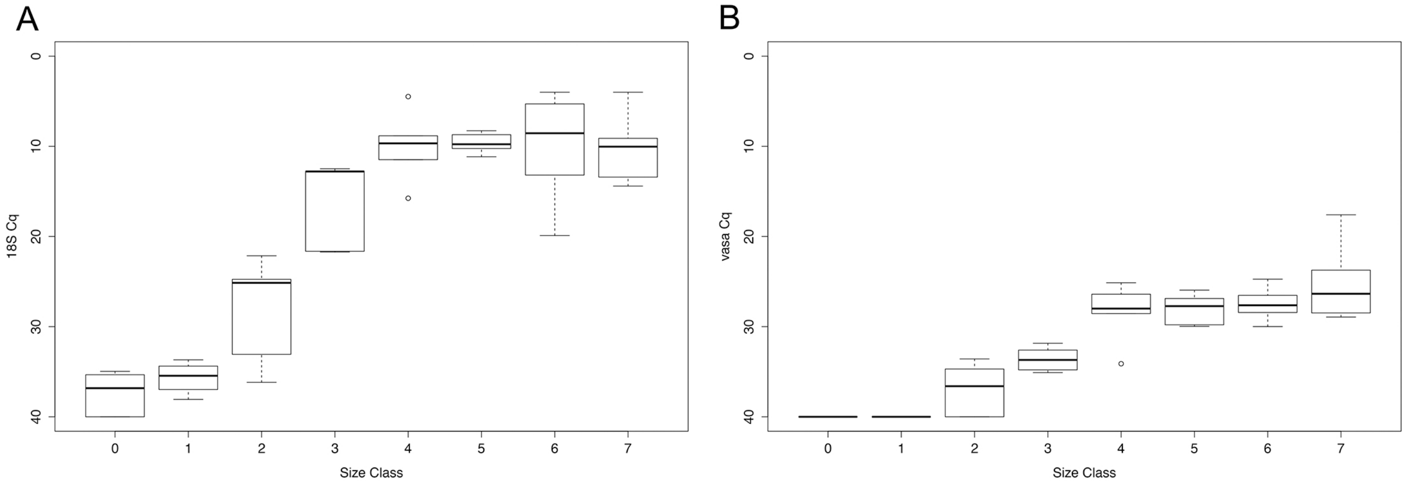

Supplement: S1 Fig — (PDF) [file pone.0137468.s001.pdf]
