# Supplementary material for: The Expression of a Novel Mitochondrially-Encoded Gene in Gonadic Precursors May Drive Paternal Inheritance of Mitochondria
Source: PLoS One. 2015 Sep 4;10(9):e0137468. doi: 10.1371/journal.pone.0137468 (PMC4560408; doi:10.1371/journal.pone.0137468)

**S2 Fig. Cluster analysis of juveniles based on *vasph* transcription level: definition of the biological classes (B0, B1, and B2).**

(A) Dendrogram of *vasph* (*vasa* homolog of *Ruditapes philippinarum*) Cq performed with Ward's minimum variance method of hierarchical agglomerative clustering;

the clustering allowed the definition of B0, B1 and B2 biological classes.

(B) Plot of the cluster analysis. Numbers represent size classes, colors represent biological classes.

The four individuals in blue were included in class B2, following the Cluster dendrogram.

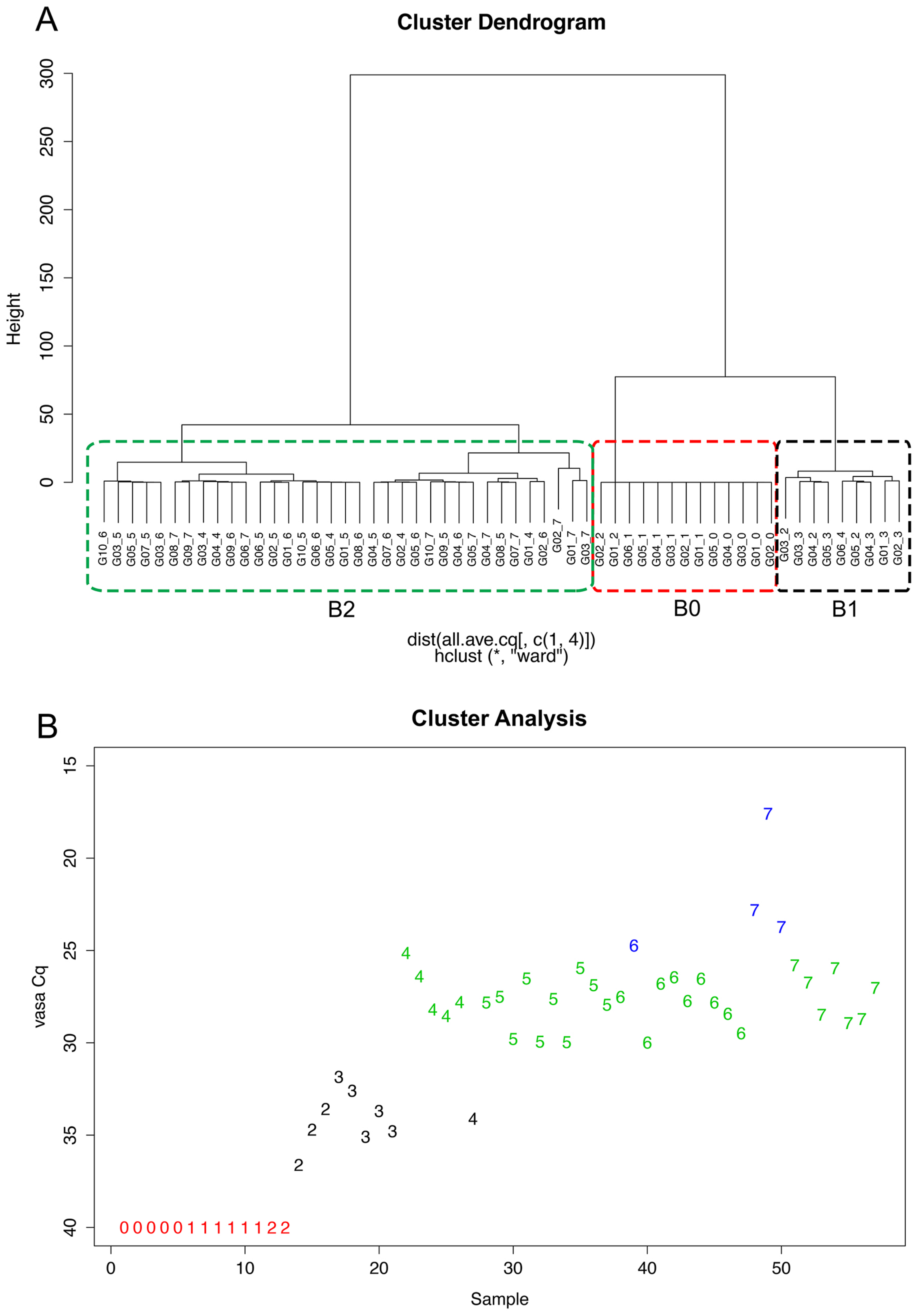

Supplement: S2 Fig — (PDF) [file pone.0137468.s002.pdf]
