# Supplementary material for: The Expression of a Novel Mitochondrially-Encoded Gene in Gonadic Precursors May Drive Paternal Inheritance of Mitochondria
Source: PLoS One. 2015 Sep 4;10(9):e0137468. doi: 10.1371/journal.pone.0137468 (PMC4560408; doi:10.1371/journal.pone.0137468)

S3 Fig. Relationships between the transcription level of *vasph* and mitochondrial targets.

Axes in juveniles: Log10 copy number ("absolute quantification" with standard dilutions).

Green dots in juveniles: biological class B1

Black dots in juveniles: biological class B2

Axes in adults: Log10 copy number relative to *18S* (relative quantification with *18S* as nuclear endogenous control).

Dashed line: equal transcription line (x=y)

Red line: regression line (shown only when the correlation is statistically supported)

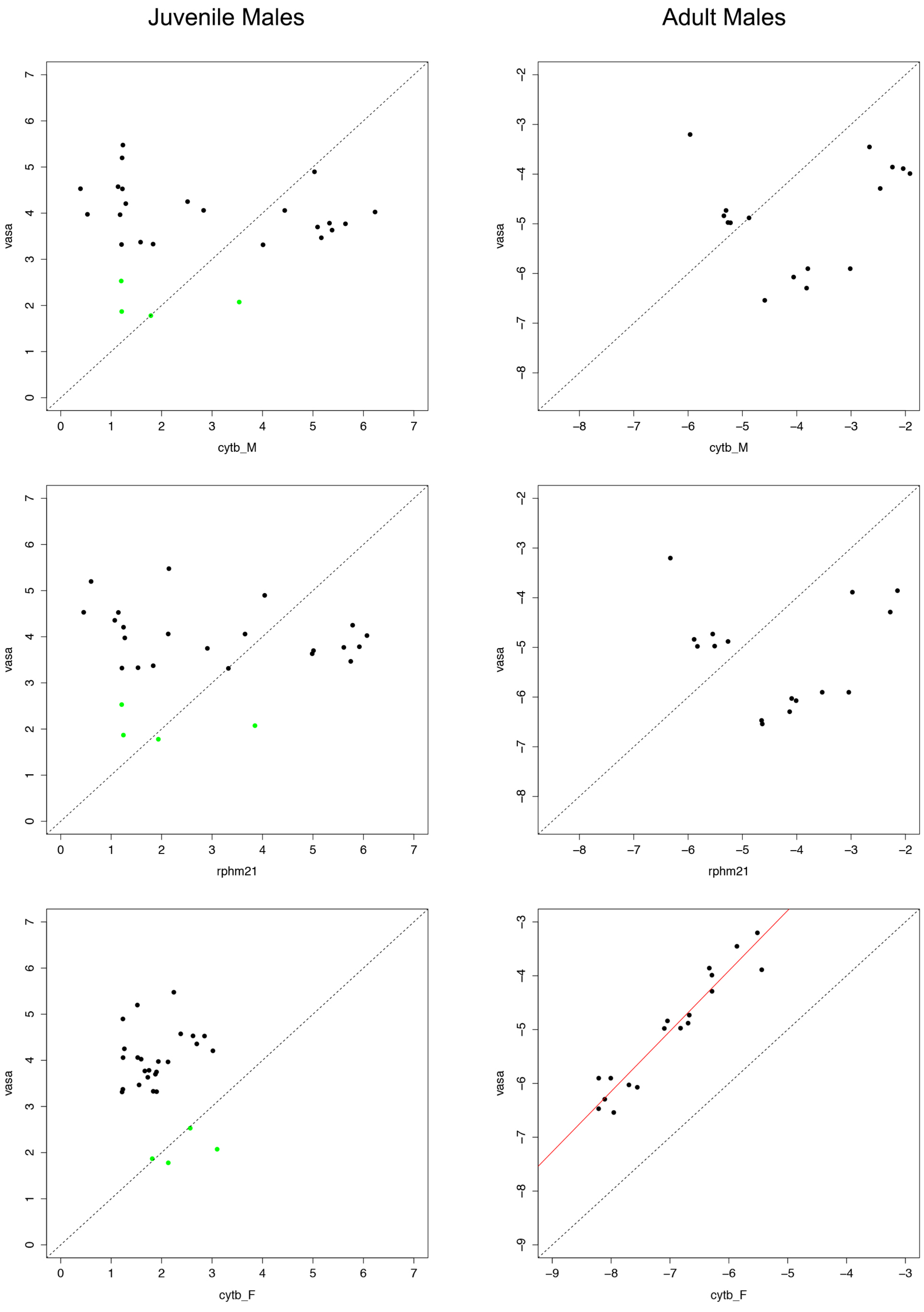

Supplement: S3 Fig — (PDF) [file pone.0137468.s003.pdf]
