# Supplementary material for: The Expression of a Novel Mitochondrially-Encoded Gene in Gonadic Precursors May Drive Paternal Inheritance of Mitochondria
Source: PLoS One. 2015 Sep 4;10(9):e0137468. doi: 10.1371/journal.pone.0137468 (PMC4560408; doi:10.1371/journal.pone.0137468)

## S5 Fig. Immunohistochemical negative controls.

Control sections of adult male (A-C) and female (D-F) tissues in which the primary antibody was omitted.

(A) Section showing a portion of male intestinal tissue (batiprismatic cells, bc), connective tissue (c), and an acinus (dashed oval) full of spermatogenic cells. Germ cells with a roundish nucleus (arrow) located inside the intestinal epithelium, as other germ cells at different stages of differentiation visible inside the acinus, result unstained. Also intestinal batiprismatic cells (bc) and connective cells (c) appear unstained. The asterisk indicates the basal lamina. (B, C) Portions of male mature acini containing unstained spermatogenic cells (sc) and spermatozoa (s). At higher magnification, the midpiece of spermatozoa results unstained (see spearheads in C, inset). (D) Section showing a portion of female intestinal tissue. All the visible cell types, germ cells with a roundish nucleus (arrow) and batiprismatic cells (bc), result unstained (magnification in the inset). (E, F) Portions of female gonadic tissue in which all cells appear unstained. In acini (dashed oval), growing oocytes with big nuclei (n) and diffused chromatin appear unstained. The TO-PRO3 nuclear dye stains all nuclei (in blue).

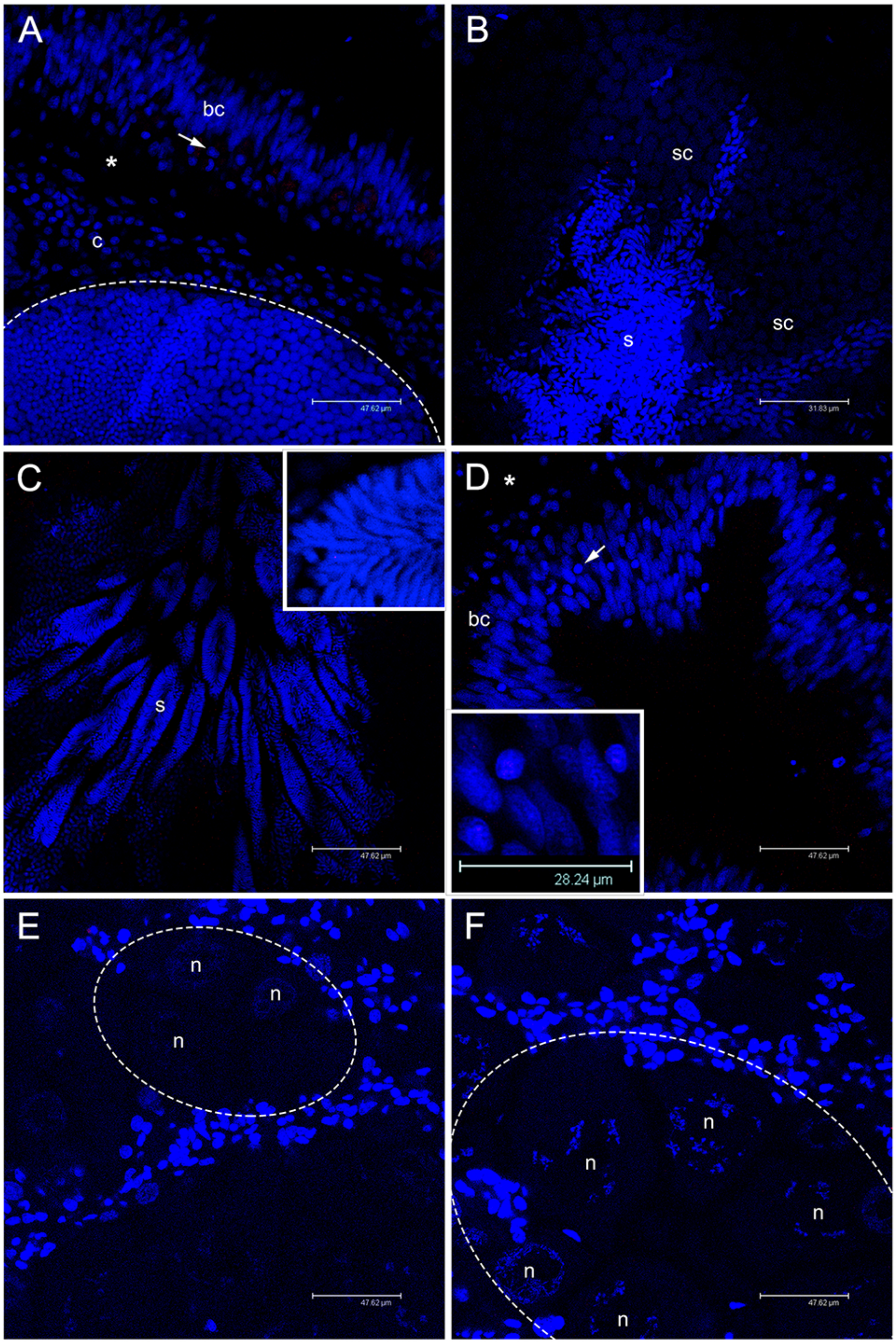

Supplement: S5 Fig — (PDF) [file pone.0137468.s005.pdf]
