# Supplementary material for: The Expression of a Novel Mitochondrially-Encoded Gene in Gonadic Precursors May Drive Paternal Inheritance of Mitochondria
Source: PLoS One. 2015 Sep 4;10(9):e0137468. doi: 10.1371/journal.pone.0137468 (PMC4560408; doi:10.1371/journal.pone.0137468)
Supplement: S1 Table — (PDF) [file pone.0137468.s006.pdf]

S1 Table. Primers used in Real-Time qPCR.

target	primer 3'-5'	product (bp)
Rph_SYBR_*18S*_ forward*	TTCAAATGTCTGCCCTATCAACTG	136
Rph_SYBR_*18S*_ reverse*	GCCTGCTGCCTTCCTTGG	
Rph_SYBR_*cytb*M_ forward*	GTTGGGATAGTTTTAATAGGTTTG	126
Rph_SYBR_*cytb*M_ reverse*	TTCGGGCTGAATATGGAC	
Rph_SYBR_*orf21*_ forward*	TCTGTGAAAGGAAACCCATGTGAG	129
Rph_SYBR_*orf21*_ reverse*	ACTAATAATAATTGGAGCCGAATAAACTTG	
Rph_SYBR_*cytb*F_ forward	GTGCCAAATAAATTAGGTGGTATTAGA	109
Rph_SYBR_*cytb*F_ reverse	CCATATTTATTCTTACACCTCGAAAAA	
Rph_SYBR_*vasa*_ forward	GCACGATTCCGACTCCGGGATG	115
Rph_SYBR_*vasa*_ reverse	GCCAGCTGTGGAAACACTGCCT	

(* from: Milani L, Ghiselli F, Iannello M, Passamonti M. 2014. Evidence for somatic transcription of male-transmitted mitochondrial genome in the DUI species *Ruditapes philippinarum* (Bivalvia: Veneridae). Curr Genet. 60:163–173.)
