# Supplementary material for: The Expression of a Novel Mitochondrially-Encoded Gene in Gonadic Precursors May Drive Paternal Inheritance of Mitochondria
Source: PLoS One. 2015 Sep 4;10(9):e0137468. doi: 10.1371/journal.pone.0137468 (PMC4560408; doi:10.1371/journal.pone.0137468)
Supplement: S2 Table — (PDF) [file pone.0137468.s007.pdf]

S2 Table. Juvenile sample for qPCR: subdivision in size and biological classes.

Individual	Biological class	mt type
J01_0	0	U
J02_0	0	U
J03_0	0	U
J04_0	0	U
J05_0	0	U
J01_1	0	U
J02_1	0	U
J03_1	0	U
J04_1	0	U
J05_1	0	U
J06_1	0	U
J01_2	0	U
J02_2	0	U
J03_2	1	m
J04_2	1	m
J05_2	1	m
J01_3	1	m
J02_3	1	M
J03_3	1	M
J04_3	1	m
J05_3	1	M
J01_4	2	m
J02_4	2	m
J03_4	2	m
J04_4	2	M
J05_4	2	M
J06_4	1	M
J01_5	2	M
J02_5	2	M
J03_5	2	M
J04_5	2	m
J05_5	2	M
J06_5	2	M
J07_5	2	M
J08_5	2	M
J09_5	2	M
J10_5	2	m
J01_6	2	M
J02_6	2	M
J03_6	2	M
J04_6	2	m
J05_6	2	m

J06_6	2	m
J07_6	2	M
J08_6	2	m
J09_6	2	M
J10_6	2	M
J01_7	2	M
J02_7	2	m
J03_7	2	M
J04_7	2	M
J05_7	2	m
J06_7	2	M
J07_7	2	M
J08_7	2	M
J09_7	2	M

Acronyms:
J = juvenile;
first number = specimen number;
second number = size class.
Note:
U = undetermined;
m = absence of M-type mtDNA;
M = presence of M-type mtDNA.
