# Supplementary material for: The Expression of a Novel Mitochondrially-Encoded Gene in Gonadic Precursors May Drive Paternal Inheritance of Mitochondria
Source: PLoS One. 2015 Sep 4;10(9):e0137468. doi: 10.1371/journal.pone.0137468 (PMC4560408; doi:10.1371/journal.pone.0137468)
Supplement: S3 Table — (PDF) [file pone.0137468.s008.pdf]

S3 Table. Summary of the juvenile samples used for the different analyses.

Size class	valve length	Sc N° qPCR	Biological class	Bc N° qPCR	valve length	Bc N° imm.
S0	1-2 mm	5	B0	13	1-5 mm	-
S1	3 mm	6				
S2	5-6 mm	5	B1	9	6-7 mm	13
S3	8 mm	5				
S4	9-15 mm	6	B2	35	8-25 mm	6
S5	18-20 mm	10				
S6	21 mm	10				
S7	25 mm	10				

Sc = size class;
Bc = biological class;
N° = number of samples used;
imm. = immunohistochemistry.
