# Supplementary material for: The Expression of a Novel Mitochondrially-Encoded Gene in Gonadic Precursors May Drive Paternal Inheritance of Mitochondria
Source: PLoS One. 2015 Sep 4;10(9):e0137468. doi: 10.1371/journal.pone.0137468 (PMC4560408; doi:10.1371/journal.pone.0137468)
Supplement: S4 Table — (PDF) [file pone.0137468.s009.pdf]

S4 Table. Transcription correlation statistics.

Group	Comparison	df	lm adj R^2	Pearson's r	p-value	Significance	Fig.
Juvenile Females	vasa - cytb_F	14	0.52	0.74	9.84E-04	***	2A
Adult Females	vasa - cytb_F	12	0.95	0.98	1.56E-09	***	2B
Juvenile Males	vasa - cytb_M	24	0.04	-0.03	8.82E-01	ns	S3
Adult Males	vasa - cytb_M	14	0.02	0.22	4.16E-01	ns	S3
Juvenile Males	vasa - rphm21	24	0.04	0.01	9.56E-01	ns	S3
Adult Males	vasa - rphm21	14	0.07	-0.04	8.88E-01	ns	S3
Juvenile Males	rphm21 - cytb_M	22	0.82	0.91	9.52E-10	***	S4
Adult Males	rphm21 - cytb_M	12	0.94	0.97	5.52E-09	***	S4
Juvenile Males	cytb_M - cytb_F	24	0.17	-0.46	1.93E-02	*	S4
Adult Males	cytb_M - cytb_F	14	0.03	0.19	4.86E-01	ns	S4
Juvenile Males	rphm21 - cytb_F	24	0.16	-0.44	2.46E-02	*	S4
Adult Males	rphm21 - cytb_F	14	0.07	-0.05	8.50E-01	ns	S4

df = degrees of freedom;
sn = non significant;
S = Supplementary figure.
